# Exploring the therapeutic potential of Aloin: unraveling neuroprotective and anticancer mechanisms, and strategies for enhanced stability and delivery

**DOI:** 10.1038/s41598-024-67397-9

**Published:** 2024-07-20

**Authors:** Stefania Zimbone, Valeria Romanucci, Armando Zarrelli, Maria Laura Giuffrida, Michele F. M. Sciacca, Valeria Lanza, Tiziana Campagna, Ludovica Maugeri, Salvatore Petralia, Grazia Maria Letizia Consoli, Giovanni Di Fabio, Danilo Milardi

**Affiliations:** 1 Istituto di Cristallografia - CNR Sede Secondaria di Catania, Via P. Gaifami 18, 95126 Catania, Italy; 2 Department of Chemical Sciences, University of Naples Federico II, Via Cintia 4, 80126 Naples, Italy; 3 Department of Drug Science and Health, University of Catania, 95125 Catania, Italy; 4 Istituto di Chimica Biomolecolare, CNR Sede Secondaria di Catania, Via P. Gaifami 18, 95126 Catania, Italy

**Keywords:** Neurodegeneration, Proteasome, Carbon dots, Amyloid, Anticancer, Biochemistry, Cancer, Plant sciences, Molecular medicine, Chemistry, Biochemistry, Chemical biology, Medicinal chemistry

## Abstract

We investigate the therapeutic potential of Aloin A and Aloin B, two natural compounds derived from *Aloe vera* leaves, focusing on their neuroprotective and anticancer properties. The structural differences between these two epimers suggest that they may exhibit distinct pharmacological properties. Our investigations revealed that both epimers are not stable in aqueous solution and tend to degrade rapidly, with their concentration decreasing by over 50% within approximately 12 h. These results underscore the importance of addressing issues such as the need for encapsulation into effective drug delivery systems to enhance stability. ThT fluorescence experiments showed that neither compound was able to inhibit Aβ amyloid aggregation, indicating that other mechanisms may be responsible for their neuroprotective effects. Next, an equimolar mixture of Aloin A and Aloin B demonstrated an ability to inhibit proteasome in tube tests, which is suggestive of potential anticancer properties, in accordance with antiproliferative effects observed in neuroblastoma SH-SY5Y and HeLa cell lines. Higher water stability and increased antiproliferative activity were observed by encapsulation in carbon dot nanoparticles, suggesting a promising potential for further in vivo studies.

## Introduction

**Figure 1:**
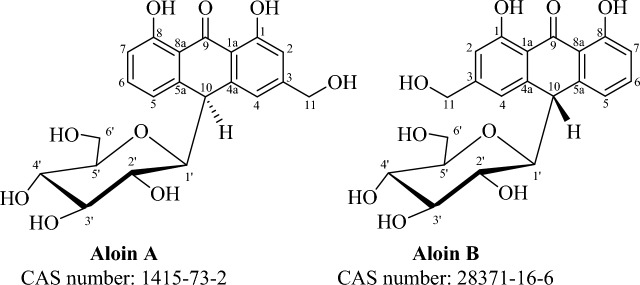
Chemical structures of Aloin A and its epimer, Aloin B, highlighting the isomeric difference in the glycosidic linkage position (10S for Aloin A and 10R for Aloin B) and their shared anthrone C-glycoside structure.

In recent years, there has been growing interest in the potential therapeutic benefits of natural compounds for various health conditions, including cancer and neurodegenerative diseases. Aloin is a bioactive compound (Fig. 1) derived from *Aloe vera* leaves that has shown some potential as a drug candidate for several diseases, including cancer and brain injury^1^. Aloin has been proposed to possess neuroprotective effects, such as improving cognitive dysfunction, preventing chronic gliosis, and exhibiting antioxidant and anti-inflammatory properties^1–7^. Recent studies have also suggested that Aloin could serve as a new neuroprotective compound and caspase inhibitor through its antiaggregating effects and activation of survival mechanisms^8^. Studies have demonstrated that Aloin activates phosphatidylinositol-3-kinase/protein kinase B (PI3K/Akt) signaling, a pathway involved in promoting cell survival and inhibiting apoptosis. By enhancing this essential pathway, Aloin confers protection against neuronal cell death and reduces the risk of neurodegenerative disorders^5^. An additional area of interest in the study of Aloin is its potential as anticancer agent. Many studies have shown that Aloin inhibits tumor angiogenesis and growth by blocking STAT3 activation, a protein involved in cell growth and survival^2,3,9–11^. Furthermore, Aloin has been found to induce cell cycle arrest and apoptotic cell death in various human cancer cell lines^2^. These findings suggest that Aloin may be a promising therapeutic option for cancer treatment^12^. Despite the promising therapeutic potential of Aloin, there are also some drawbacks to consider when using it in therapy. Firstly, the exact mechanisms through which Aloin exerts its neuroprotective and anticancer effects are not fully understood. More research is needed to determine the specific pathways and targets involved. In addition, many studies do not consider the fact that Aloin (AloAB) is a mixture of two isomers Aloin A (barbAloin, AloA) and Aloin B (isobarbAloin, AloB), which have an anthronic C-glycoside structure with different stereochemistry at their C-10 (Fig. 1)^13^. In AloA the configuration at C10 is S, while in AloB is R. The aloe-emodin anthrone consists of three rings: two are aromatic and the third is a cyclohexan-9-one ring. The difference in positioning of the glycosidic linkage makes them epimers at C-10, resulting in their different physical and possibly pharmacological properties^14,15^.

Notably, the structural configuration can deeply impact the absorption, distribution, metabolism, and excretion of these compounds in the human body. Therefore, considering the structural differences of these molecules is critical for studying their biological effects. Additionally, the bioavailability and pharmacokinetics of Aloin need to be further investigated to optimize its delivery and ensure effective therapeutic concentrations in target tissues. As for many other natural compounds, Aloins are not stable molecules and rapidly degrade over time, which may limit their shelf-life and efficacy in therapeutic applications^16^. We will assess the stability of AloA and AloB in aqueous solutions, examining potential degradation into diverse end-products. This aims to establish a timeframe for attributing observed effects to a specific compound. Additionally, tube tests will be employed to study the impact of these epimers on the aggregation of the amyloid Aβ peptide and the 20S proteasome, crucial therapeutic targets for neurodegeneration and cancer, respectively. Following the experimental results, we will explore the encapsulation of Aloin in targeted drug delivery systems like carbon dots (CDs)^17,18^ to enhance water stability. Subsequently, we will evaluate the potential of encapsulated drugs to influence proliferation of several cancer cell lines.

## Results and discussion

### Stability and time-dependent behavior of Aloin A and Aloin B in aqueous solutions

**Figure 2:**
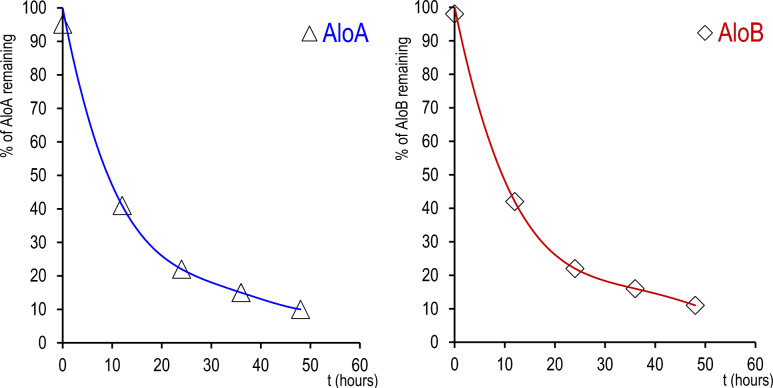
Stability of Aloin A (Δ) and Aloin B (◊) at 37 °C in PBS at pH 7.4: % Aloin remaining in the 0–48 h range.

The therapeutic potential of Aloins, despite reported benefits in numerous studies, is constrained by their poor stability and rapid degradation in aqueous solutions. Prior research has investigated the stability of Aloins and other anthraquinones across diverse pH and temperature conditions, revealing that dehydration could notably enhance their stability during storage^19^. Nevertheless, there is limited information regarding the stability of Aloin in aqueous solutions over time. To address this gap, we followed by RP-HPLC analysis, the disappearance of the peak relating to the metabolite over time (Fig. 2). AloA and AloB (purity ≥ 98%) were dissolved in PBS at pH 7.4 with 0.5% DMSO and kept in the dark, both at RT and at 37 °C, and analysed over 48 h. In agreement with the data reported^20^, through LC–MS analysis we observe for both metabolites an interconversion of AloA (t_*R*_ = 14.9 min) into AloB (t_*R*_ = 15.3 min) or vice versa after 24 h (see Figs. S1, S2 in the Supporting Information). Furthermore, the formation of the 10-OH derivative (t_*R*_ = 13.5 and 14.1 min) as the main product was observed by HPLC–MS analysis after only a few hours, for both Aloins (Figs. S3, S4 in the supporting Information). We found a persistence of AloA and AloB lower than 40% already after 12 h, which is reduced to values lower than 20% after 24 h.

These findings collectively indicate that the validity of experiments in aqueous solutions is only guaranteed within a brief timeframe, typically a couple of hours and that enhancing the stability of these compounds in water is critical for their therapeutic application.

### Exploring the role of Aloin A and Aloin B in modulating Aβ peptide amyloid aggregation

**Figure 3:**
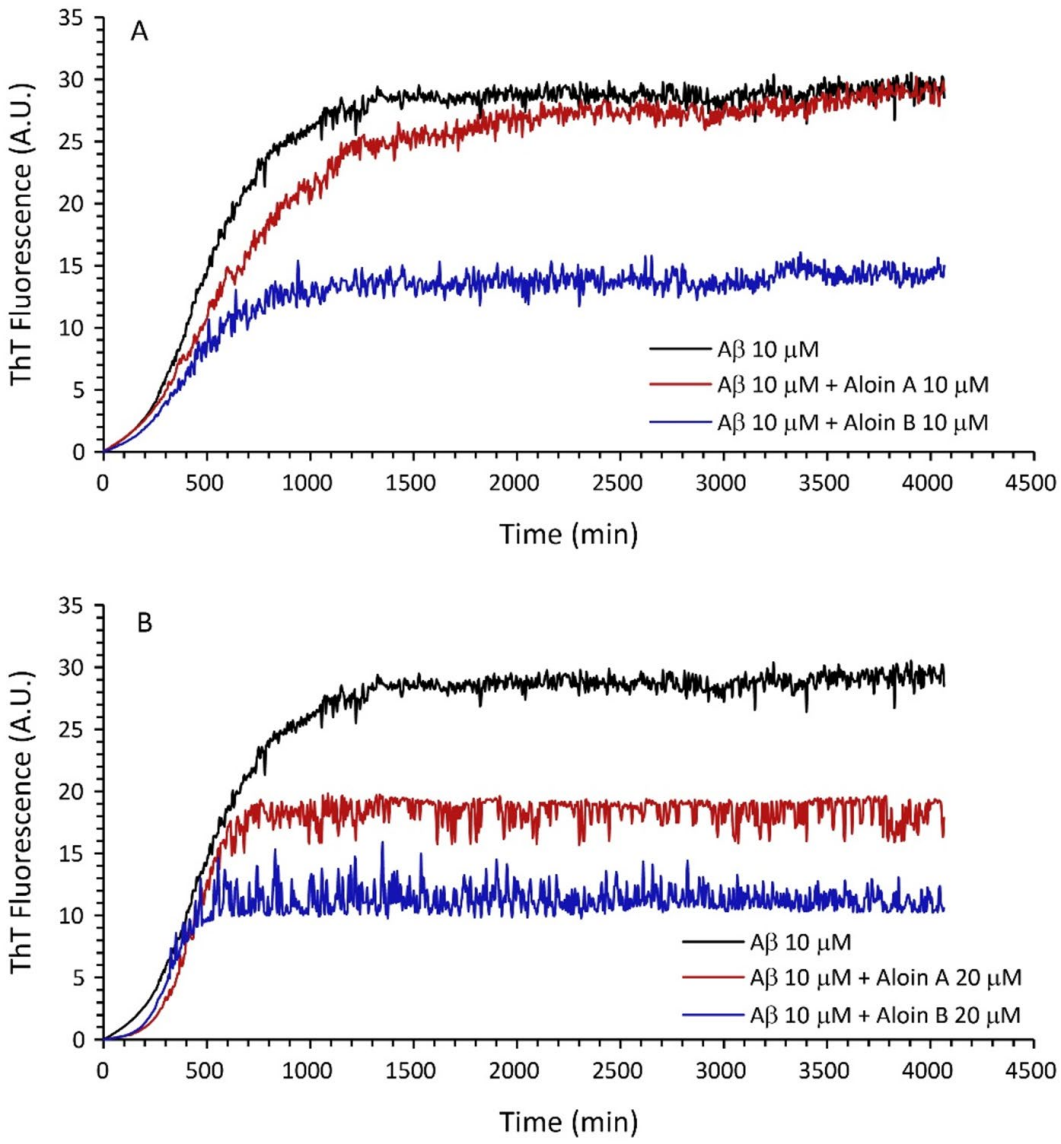
(**A**) Kinetics of fiber formation measured by ThT fluorescent emission for 10 μM Aβ1-40 (black line) and in presence of Aloin A at 1:1 ratio (red line) or Aloin B 1:1 ratio (blue line). (**B**) Kinetics of fiber formation measured by ThT fluorescent emission for Aβ1-40 10 μM (black line) and in presence of Aloin A at 1:2 ratio (red line) or Aloin B 1:2 ratio (blue line). All the experiments were conducted in phosphate buffer 10 mM, 100 mM NaCl, pH 7.4 at 37 °C. Traces are the average of three independent experiments.

Alzheimer’s disease (AD) stands as the prevailing type of age-related dementia, marked by a gradual decline in memory and cognitive functions^21^. A prominent pathological feature of the disease is the existence of extracellular aggregates of amyloid β (Aβ) peptides in senile plaques^22^. Within the AD brain, aberrant processing of the amyloid precursor protein (APP) by β- and λ-secretases can lead to high levels of Aβ peptides, ultimately accumulating in neuronal tissues and contributing to cell death^23^. Therefore, targeting the inhibition of Aβ self-assembly emerges as a promising therapeutic strategy for AD treatment^8^ and numerous compounds, including those derived from natural sources, have been evaluated for their anti-aggregating properties^24–28^. Despite exhibiting neuroprotective properties, the impact of Aloins on Aβ peptide amyloid aggregation remains unexplored. To address this gap, we investigated the influence of AloA and AloB on amyloid growth using Thioflavin T (ThT) fluorescence assays at a 1:1 Ligand:peptide molar ratio (Fig. 3). Interestingly, within a two-hour timeframe, during which Aloin molecules maintain stability in aqueous solution, their effect on Aβ aggregation is negligible. However, at longer durations, potentially involving Aloin degradation byproducts, AloB (see Fig. 3 panel A, blue curve) significantly reduces the total fiber formed, evident in the decrease in I_max_ value (see Table 1). Aloin A under these conditions (see Fig. 3 panel A, red curve) doesn’t affect any kinetic parameters significantly (see Table 1). At higher concentrations, AloB (see Fig. 3 panel B, blue curve) exhibits no concentration-dependent behavior, with a reduction in amyloid aggregates similar to that observed at a 1:1 ratio. Conversely, AloA (see Fig. 3 panel B, red curve) in a 2:1 molar ratio significantly diminishes the amount of Aβ fiber formed, indicated by a notable decrease in I_max_ (Table 1). Our findings evidence that both AloA and AloB are unable to prevent Aβ fiber formation and suggest that this mechanism is likely not involved in the neuroprotective properties of Aloins.

**Table 1:** Kinetic parameters derived from ThT curves.

Sample, concentration	I_max_ (A.U.)	t_1/2_ (min)	K_app_ (min^−1^)
Aβ1–40, 10 μM	30.2 ± 0.1	495 ± 8	5.5 × 10^–3^ ± 2.2 × 10^–4^
Aβ1–40, 10 μM Aloin A, 10 μM	30.5 ± 0.2	579 ± 15	3.4 × 10^–3^ ± 1.7 × 10^–4^
Aβ1–40, 10 μM Aloin A, 20 μM	18.6 ± 0.2	432 ± 12	1.2 × 10^–2^ ± 1.4 × 10^–3^
Aβ1–40, 10 μM Aloin B, 10 μM	14.7 ± 0.1	422 ± 17	6.0 × 10^–3^ ± 5.6 × 10^–4^
Aβ1–40, 10 μM Aloin B, 20 μM	11.3 ± 0.2	323 ± 18	1.5 × 10^–3^ ± 3.7 × 10^–4^

ThT kinetic curves were analyzed by using a sigmoidal curve in which I_max_ = maximum fluorescence intensity, t_1/2_ = time to half, τ = elongation time constant. The apparent time constant k_app_ is given by 1/τ.

### Aloin A and Aloin B are 20S proteasome inhibitors

**Figure 4:**
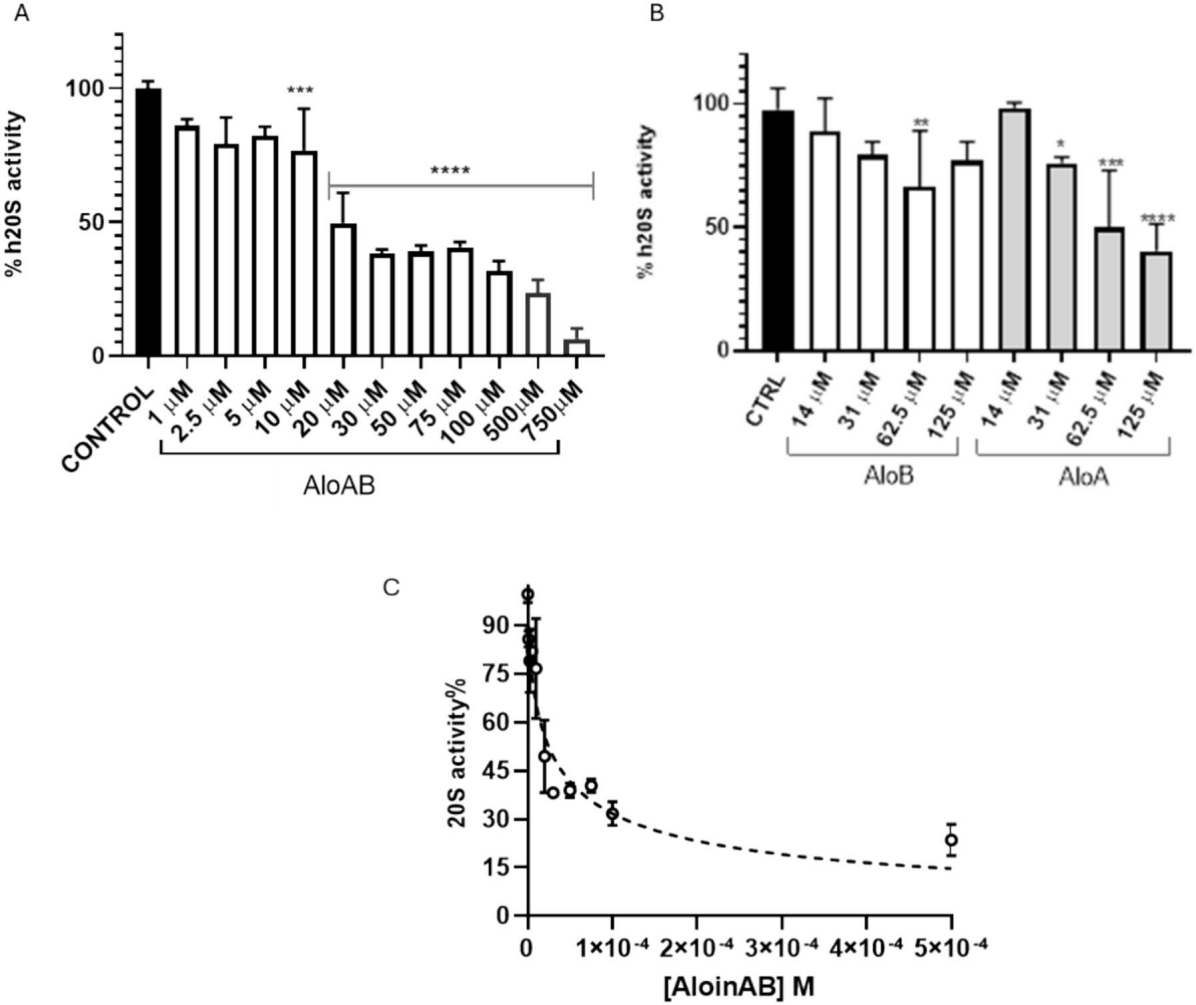
(**A**) Normalized activity of human 20S proteasome compared to the control, in an equimolar mixture (AloAB): concentration range 1 × 10^–6^–5 × 10^–4^ M. (**B**) Normalized activity of human 20S proteasome compared to the control of individually isomer AloA and AloB. (**C**) Nonlinear fit of the concentration–response plot for the inhibition of ChT-L residual activities of human 20S proteasome in the presence of increasing concentrations of AloAB.

The proteasome, a 2500 kDa proteolytic molecular machine, encompasses various enzymatic activities (proteolytic, ATPase, de-ubiquitinating) collaboratively aiming for protein degradation^29^. In eukaryotes, it consists of the 20S proteasome core particle, a cylinder-shaped multimeric protein complex capped on both ends by the 19S complex, responsible for substrate recognition, unfolding, and translocation into the 20S proteasome’s lumen^30^. This 700 kDa, cylinder-shaped protease comprises 28 protein subunits arranged in four stacked rings, each consisting of seven subunits^31,32^. Mammalian proteasomes exhibit five different peptidase activities: chymotrypsin-like (ChT-L), trypsin-like (T-L), peptidylglutamyl-peptide hydrolyzing (PGPH), branched-chain amino acid preferring (BrAAP), and small neutral amino acid preferring (SNAAP). These activities cleave bonds on the carboxyl side of hydrophobic, basic, acidic, branched chain, and small neutral amino acids, respectively^33^. According to proteolysis models, subunits in the 19S particle recognize polyubiquitin chains of four or more residues on the target protein; other subunits cleave the polyubiquitin chain for recycling. Target proteins undergo unfolding and are directed into the 20S proteolytic core for cleavage into small peptides. These peptides are released from the proteasome and degraded into amino acids by cytosolic exopeptidases. Several preclinical studies have documented enhanced proteasome expression and activity across various cancer types^34–38^. The underlying cause of augmented proteasome activity remains unclear, although it is likely associated with stressful conditions within the tumor microenvironment. The inhibition of proteasome activity has hence the potential to disturb the balance between tumor suppressors and oncoproteins, thereby decreasing cancer progression^39–41^. While the anticancer properties of Aloins targeting various mechanisms are established, their impact on the proteasome remains unexplored. To address this issue, Aloins effects on proteasome 20S activity were assessed using a fluorogenic peptide to measure ChT-L peptidase activities, as detailed elsewhere^42﻿^. The fluorescence intensity over time was plotted, with the slope indicating the inhibition potency. In these assays, the equimolar mixture of AloA and AloB (AloAB) and the pure epimers AloA and AloB were tested in the same range of concentration (1–750 µM). Increasing concentrations of Alo AB exhibited a more pronounced inhibitory effect compared to equivalent concentrations of pure isomers AloA and AloB (Fig. 4, panel A and B). This behavior can be explained by considering that, because the structures of the two molecules differ, they may occupy distinct allosteric sites that work in concert to increase the overall effect. This hypothesis, however, should be confirmed by further structural studies of ligand-target complexes. Encouraging and reproducible data were observed with Alo AB and human 20S proteasome, displaying a significant decrease in chymotryptic activity at concentrations exceeding 5 µM. The IC_50_ value for Alo AB (28 µM ± 7) was calculated as described in the experimental section, and the normalized concentration–response plot is shown in Fig. 4, panel C. These findings indicate that the combination of the two Aloins could serve as a potent proteasome inhibitor, potentially possessing antiproliferative properties.

### CDs-PNM/AloAB adduct preparation and characterization

**Figure 5:**
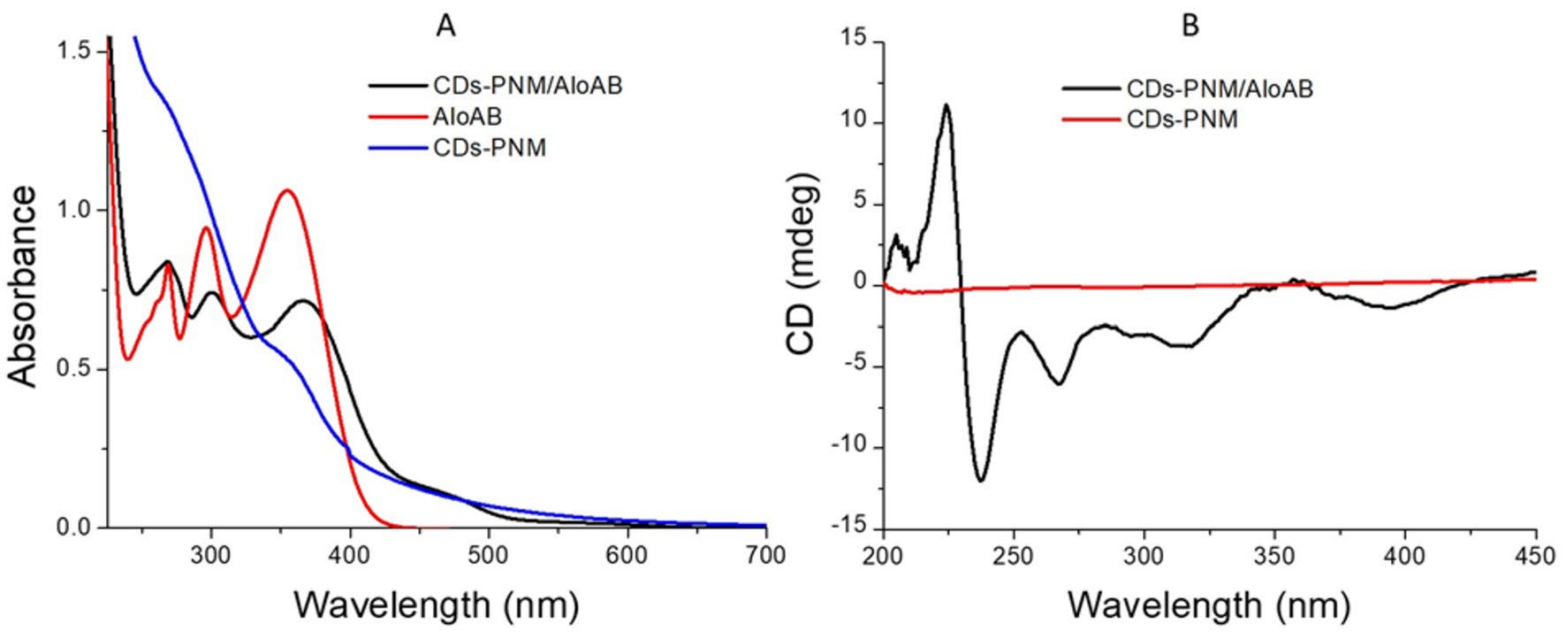
CDs-PNM/AloAB spectroscopical measurements: (**A**) optical absorption spectra of CDs–PNM 10 µg/µL (blue-line), Aloin-loaded CDs–PNM (black line) in water and AloAB in water (red line) and (**B**) CDs spectra of CDs-PNM/AloAB and CDs-PNM.

Carbonized polymer dots (CDs), composed of an inner carbonized core covered by a polymer chain shell, have received significant interest in the field of nanotechnology for biomedical application including drug delivery. Recently, we developed a novel one-pot synthetic strategy to produce biocompatible, luminescent and photothermally responsive carbon polymer dots starting from poly(*N*-isopropylacrylamide) (PNM)^43,44^. The carbonized polymer dots-PNM (CDs–PNM) were obtained by simple heating of PNM (200 °C, 4 h), without using solvents and additives, through a condensation/aromatization mechanism. Atomic force microscopy (AFM) and transmission electron microscopy (TEM) analyses and dynamic light scattering measurements evidenced the spherical shape and nano-dimensions of the CDs–PNM (diameter around 100–200 nm with a carbon-core of 8 ± 2 nm)^43,44^. We demonstrated that the CDs–PNM possess drug loading/release properties controlled by a lower critical solution temperature (LCST) behavior, that induces a coil-to-globule conformational transition of the polymer chains. Practically, at room temperature (25 °C) the CDs-PNM adopt a hydrophilic coil-structure permitting an effective drug loading process while at higher temperature (37 °C) the transition to a hydrophobic globule-structure induces an efficiently drug release effect. This mechanism was largely demonstrated for various cargo molecules as curcumin^43,45^, methylene blue^46^ cytarabine^45^ and doxorubicin^47^. Basing on the demonstrated drug loading versatility and drug delivery potentialities^45^, we decided to investigate the CDs–PNM as a novel nanocarrier for AloAB. The loading of AloAB in the CD–PNM nanocarrier could results in an increased stability of this noticeably unstable natural product and improved intracellular vehiculation due to the demonstrated cellular uptake of the CDs-PNM^43^. The aqueous dispersion of CDs-PNM/Alo AB was prepared as described in the experimental section. The AloAB loading capacity percentage (LC%) was calculated to be about 33.3% and drug release at 37 °C is ascribable to the coil-to-globule conformational transition, as previously reported for other CDs-PNM/drug adducts. The CDs-PNM/AloAB adduct formation was confirmed by spectroscopic and DLS measurements. In details, the UV–Vis absorption spectrum of CDs-PNM/AloAB showed the typical AloAB absorption bands at 268 nm, 300 nm and 366 nm, red-shifted compared to the absorption bands of AloAB in water (268 nm, 296 nm and 355 nm) (Fig. 5A). The n–π* broad-band at 360 nm and the π–π* at 271 nm confirmed the presence of the CDs core in the CDs-PNM/AloAB adduct. Similarly, the Circular Dichroism (CD) measurements supported the CDs-PNM/AloAB nanostructures formation through the presence of diagnostic signals in the range 230–310 nm (Fig. 5B), typical of AloB and AloA (Fig. S5 of Supporting Information). No CD signal was recorded for the CDs-PNM water dispersion.

**Figure 6:**
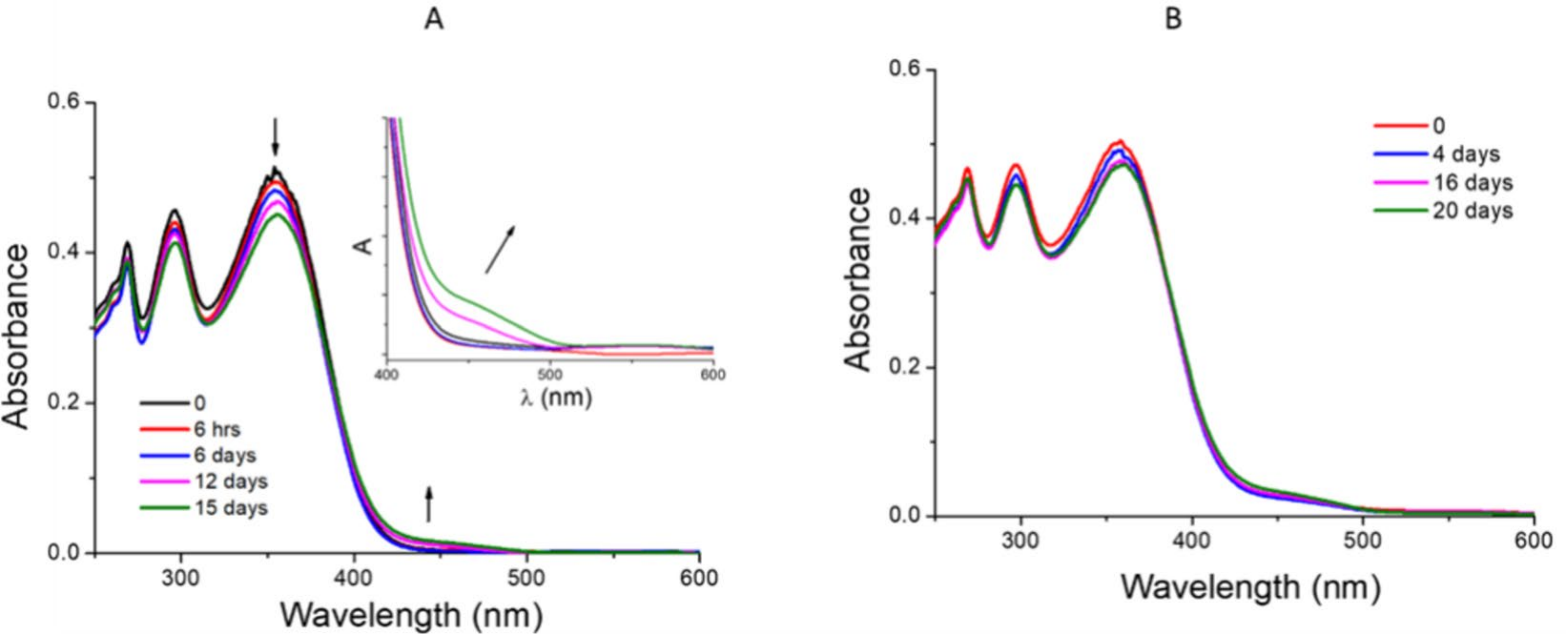
Optical absorption changes of (**A**) Alo B and (**B**) CDs-PNM/AloAB in water, after storage in the dark at room temperature.

DLS measurements for CDs–PNM/AloAB in water at pH 7.0 and temperature of 25 °C showed a main population with mean hydrodynamic diameter centered at 246 ± 10.1 nm, greater than the hydrodynamic size obtained for CDs–PNM (101 ± 9 nm). The Z-potential investigation showed the presence of negatively charged (− 13.5 ± 0.35 mV) nanostructures. According to a previous work,^44^ our findings indicate that CDs-PNM nanostructures exhibit a protective action against the degradation of AloAB in water. The optical absorption changes, monitored over time (0–15 days) for AloAB and CDs-PNM/AloAB water solutions stored at room temperature and in the dark (Fig. 6A,B), show that the absorption of AloAB at 355 nm decreases by 12% after 15 days whereas the corresponding band of CDs-PNM/AloAB at 360 nm decreases by 6% after 16 days. At the same time, the increase of the absorbance band at 450 nm, related to AloAB degradation, was 5 times lower in the presence of CDs-PNM furthering supporting the stabilization effect.

### Antiproliferative activity of Aloins and Aloins-loaded CD nanoparticles

**Figure 7:**
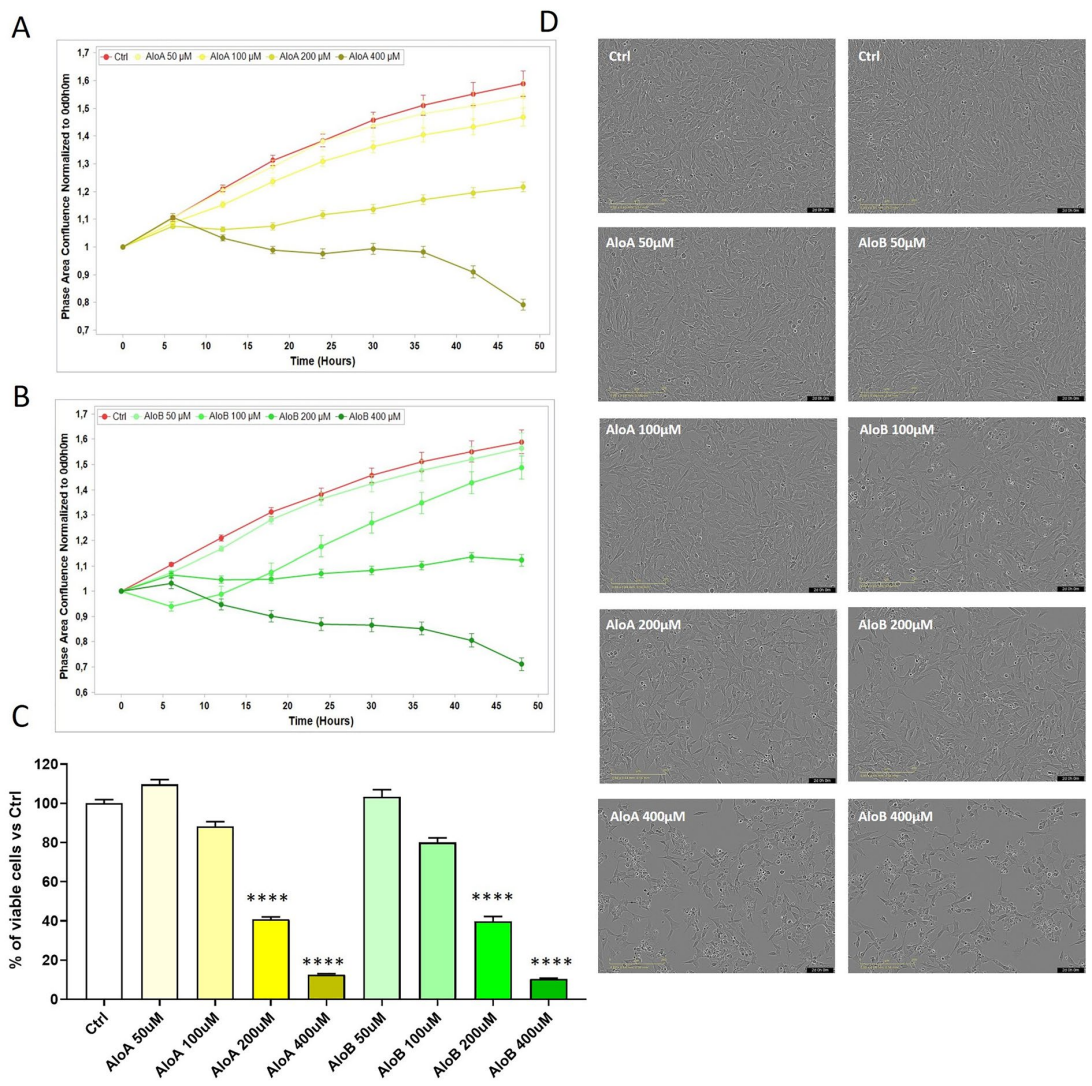
Antiproliferative response in neuroblastoma SH-SY5Y cells to increasing concentrations (50–400 μM) of AloA (**A**) and AloB (**B**) as quantified by the Incucyte SX1 Live-Cell Analysis System. The readout of cellular growth was assessed every 6 h over 48 h. Values for each timepoint represent means ± SEM of 3 replicates and are normalized to control wells. (**C**) MTT analysis of the neuroblastoma cell lines treated with Alo A and Alo B. Cell viability was assessed after 48 h of treatment. Bars represent means ± SEM of three independent experiments with n = 3 each. ****P < 0.0001, versus Ctrl by one-way ANOVA + Dunnett’s test. (**D**) Representative optical images of human neuroblastoma cells after 48 h of exposure with AloA or AloB (50–400 μM).

**Figure 8:**
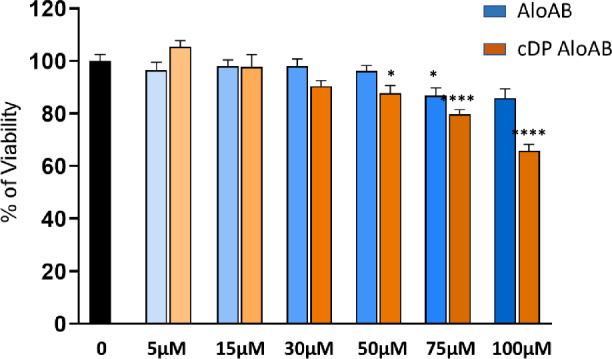
MTT analysis of the neuroblastoma cell lines treated with CDPs-PNM (0.2 mg/mL) alone or loaded with increasing concentrations of AloAB (5–100 μM). Cell viability was assessed after 48 h of treatment. Bars represent means ± SEM of three independent experiments with n = 3 each. ****P < 0.0001, *P < 0.05 versus Ctrl by one-way ANOVA + Tukey test.

The isolation of the two epimers AloA and AloB from the *Aloe Vera* leaves allows the investigation of the biological activities of the two compounds, separately. Among the recognized properties of *Aloe Vera*, we chose to study the antiproliferative activities of AloA and AloB, on human neuroblastoma cell line SH-SY5Y. Based on previous data^18,48,49^ we exposed the cells to concentrations ranging from 50 to 400 µM, and we tested the antiproliferative effect of the treatments with the Incucyte SX1 Live-Cell Analysis System. After 48 h of exposure, using the Basic Analyzer Software, we obtained a readout of cytotoxicity over time in a label-free manner and automatically measured within the cellular incubator on living cells (Fig. 7A,B). The growth curves revealed a similar trend for all the treatments applied. Indeed, as expected, untreated cells showed exponential cellular growth over time, starting from 1, which was arbitrarily assigned to the initial cell seeding density, to about 1.5 after 48 h of incubation. These values correspond to the phase area confluence normalized to Time 0. On the contrary, a dose–response effect was observed for both Alo A and Alo B. In particular, exposure to 50 and 100 µM of single compounds, did not affect significantly the normal rate of growth, while 200 µM and 400 µM concentrations, were able to reduce the rate of growth and cellular confluence to 0.7 after 48 h of treatment, as also indicated in Fig. 7. Results were further validated through MTT assays (Fig. 7C), ensuring consistency in the measurement of cell viability under identical conditions. In summary, the data implies that while separating the two epimers may be advantageous for evaluating a robust structure–activity relationship, taking also into account the influence of principal metabolites like emodin^50^, the tested biological activity does not indicate any discernible differences between Aloin A and B. The antiproliferative activity observed for AloA and AloB was then compared to AloAB which showed a similar extent of effect in the dose–response experiment (Fig. S6 of Supporting information) as confirmed by the IC_50_ values calculated for each curve (AloA:213 ± 33.3; AloB:198.7 ± 31; AloAB:218.9 ± 38.9). To further validate the obtained results, we used HeLa as a different model of a cancer cell line. Data revealed a more resistant behavior of these cells to Aloin compared to SH-SY5Y (Fig. S7 of supporting information). Accordingly, under the same conditions, the proteasome inhibitor, bortezomib, showed higher efficacy on neuroblastoma cells compared to HeLa (see IC_50_ values in Fig. S8 of Supporting information), suggesting that neuroblastoma cells can be more sensitive to compounds with proteasome inhibitory activity. Given that the epimers AloA and AloB exhibited similar effects on neuronal cells and to improve the slight antiproliferative activities of the compounds, we loaded AloAB onto carbon-based polymer dots (CPDs-PNM), which are known to be safe for cells^43^ and suitable carriers for drugs with poor water-solubility and bioavailability. We compared the antiproliferative effect of CPDs-PNM/AloAB with the activity of AloAB alone, on human neuroblastoma cells using a lower range of concentrations (5–100 μM). As shown in Fig. 8, AloAB loaded in CDs-PNM, showed greater antiproliferative activity, compared to the free compound. Specifically, at 100 μM concentration, 65% of cell viability was observed by MTT assay after 48 h of treatment. The higher cytotoxic effect of the CDs-PNM/AloAB may be related to the preservation of the entrapped Aloin from degradation and/or more effective cellular uptake already demonstrated for the luminescent CDs-PNM on neuroblastoma cells^44^.

## Conclusions

In this study, we explored the therapeutic potential of two derivatives extracted from the *Aloe vera* leaves called Aloin A (AloA) and Aloin B (AloB). The separation of the two epimers aimed to establish a structure–activity relationship and the stability of these compounds in water, a critical consideration given the aqueous nature of many pharmaceutical or nutraceutical preparations. Our investigations revealed that both epimers are not stable in neutral aqueous solution and tend to degrade rapidly, with their concentration decreasing by over 50% within approximately 12 h. This information is crucial not only for assessing product efficacy in prevention or therapy but also for validating experimental data obtained in studies employing an incubation period exceeding 12 h. As a consequence, all experiments deemed valid by us were conducted under conditions ensuring that observed effects could be attributed to Aloin rather than its degradation products. While existing literature highlights the neuroprotective effects of *Aloe vera* extracts, there was a lack of data regarding their ability to inhibit amyloid aggregation. Our ThT fluorescence studies demonstrated that both products were not significantly effective in inhibiting amyloid aggregation, suggesting that neuroprotection mechanisms may be attributed to their antioxidant properties or other mechanisms. Regarding their anticancer properties, we observed a significant ability of the equimolar mixture of AloA and AloB to inhibit proteasome activity in tube tests. Given the known antitumor effects of proteasome inhibitors, we evaluated the antiproliferative activity of Aloins on SH-SY5Y and HeLa cell lines. The data showed that both AloA and AloB exhibited similar antiproliferative activities under identical conditions. Based on these results, we encapsulated these mixtures in carbon dot nanoparticles to enhance stability over time and improve their ability to penetrate cell membranes, thereby optimizing their biological activity. Encapsulation studies yielded positive outcomes, demonstrating that  the compounds encapsulated within nanoparticles exhibited far greater stability over time in aqueous solution compared to free molecules. Furthermore, the antiproliferative activity of these encapsulated Aloins in carbon dots was significantly higher. The collective results suggest that, albeit this work is only a preliminary exploration of the effects of these molecules and their mechanism of action needs to be further investigated, the use of CDs nanoparticles can enhance the stability and anticancer activity of equimolar mixtures of Aloin, making them promising candidates for further in vivo studies.

## Experimental section

### Chemicals

AloA and AloB analytical standards were purchased from Sigma-Aldrich, while HPLC-grade ACN and CH_3_OH were obtained from Carlo Erba Reagents and Sigma-Aldrich (Milano, Italy), respectively. GenScript supplied Amyloid β peptide 1–40 (Aβ) with a purity exceeding 95%. Thioflavin T (ThT) was purchased from Sigma-Aldrich, and Hexafluoro isopropanol (HFIP) was obtained from Carlo Erba. Human 20S proteasome was obtained from Boston Biochem, Inc. (Cambridge, MA). The fluorogenic substrate releasing fluorescent 7-amino-4-methylcoumarin (AMC) for the measurement of chymotrypsin-like (ChT-L) peptidase activity was succinyl-LeuLeuValTyr-AMC (SucLLVY-AMC) (Bachem Bioscience Inc.). Bortezomib was purchased from Millipore (Milano, Italy). All reagents for CDs-PNM preparation were purchased from Sigma-Aldrich (Milano, Italy). All solutions were prepared using ultrapure Thermo Milli Q water.

### Extraction of AloA and AloB from *Aloe* arborescens

Thirteen young *Aloe vera* leaves, weighing between 500 and 950 g, were collected at the Botanical Garden of Naples and identified by prof. Antonino Pollio of the Department of Biology of the Federico II University of Naples. The leaves were placed vertically with the basal part at the top and the terminal part was cut off to obtain the latex, collected over 5 h. The latex obtained (385 g) was freeze-dried weighed (39.2 g) and then a part was taken up with a PBS at pH 3.0. The solution obtained was sonicated for 12 min and then centrifuged at 5000 rpm for 5 min. The solution was separated by preparative HPLC (Shimadzu LC-8A system using a Shimadzu SPD-10A VP UV–Vis detector (Shimadzu, Milan, Italy) using a reversed-phase column Phenomenex Gemini NX-C18 (250 × 21.2 mm) 5 μm and eluted with a gradient of TFA (A, 0.02% in H_2_O) and CAN (B), starting with 15% B for 3 min and followed by the installation of a gradient to obtain 80% B over 25 min, at a solvent flow rate of 3.5 mL/min to yield AloB and AloA (Detector: 260 nm; room temperature, with injection volumes of about 1500 mL). All experimental studies on plants were carried out in accordance with relevant guidelines. The structure elucidation of AloA and AloB was achieved by 1D and 2D NMR analysis (^1^H, ^13^C, COSY, HSQS, HMBC) and the signal assignment is reported in the Supplementary Information file (see Tables S1, S2).

### Stability of compounds in PBS at pH 7.4

The stability of the two extracted and purified metabolites, AloA and AloB (approximately 98% purity) was evaluated by RP (18)-HPLC analysis over 48 h at 37 °C in PBS buffer at pH 7.4. Briefly, two stock solutions of AloA and AloB (ca 5 mg/mL, ca 12 mM) were prepared in PBS at pH 7.4 and were kept in the dark at 25 and 37 °C. 10 μL were taken at different times (0, 12, 24, 36 and 48 h) and diluted to 100 μL and 20 μL immediately injected. The analysis was conducted on an Phenomenex InterClone® RP-18 column (150 × 4.6 mm). The binary mobile phase was composed of water with 0.5% acetic acid (A) and CAN (B). The system was run with a gradient programme at 1.0 mL/min, 5–100% (B) in 30 min. The elution was monitored at 260 nm. LC–MS analysis was performed in positive ionization mode on an Agilent LC–MS ESI-TOF instrument; the following conditions were adopted: capillary voltage 3500 V; drying gas (nitrogen) 5 L/min, 325 °C; fragmentor voltage 175 V; nebulizer pressure 35 psi. Analysis was performed on an Agilent Eclipse Plus ODS column (150 × 4.6 mm, 5 μm) at a flow rate of 0.4 mL/min; the gradient elution was as follows: 0.1% formic acid (solvent A)/methanol (solvent B); 5% B, 0–10 min; from 5 to 80% B, 10–30 min.

### CDs-PNM preparation

Biocompatible and water-dispersible nanosized carbondots (CDs-PNM) were prepared by thermal treatment from poly(*N*-isopropylacrylamide) (PNM) using a one-pot method developed in our laboratories^43,44^. In detail, an amount of 50 mg of PNM was heated at 200 °C for 4 h to obtain the carbon-based nanomaterial. The brown-reddish product was washed with 1 mL of Milli-Q water. The aqueous dispersion, after sonication for 2 min, separation by centrifugation (8000 rpm 15 min) and filtration (0.2 μm pore size), was purified by dialysis through a dialysis membrane (10 kDa cut-off) for 46 h using Milli-Q water.

### Aβ monomerization

Aβ peptides were initially dissolved in HFIP at a concentration of 1 mg/mL. Subsequently, these solutions were portioned into 100 μL aliquots, frozen at − 80 °C, and subjected to lyophilization. The quantification of these aliquots was carried out by reconstituting them in 20 μL of NaOH (1 mM), which was then added to a 180 μL buffer solution (10 mM phosphate, pH 7.4). The absorbance at 280 nm was measured for quantification, using an extinction coefficient (ε = 280 nm) of 1450/M/cm.

### ThT assays﻿

For ThT assays, samples were prepared by adding 1 μL of AloA or AloB stock solutions in water (final concentrations of 0.5 μM, 2 μM, and 4 μM) to 50 μL of ThT solution in PBS (phosphate 10 mM, pH 7.4, and 100 mM NaCl, at a concentration of 20 μM). These experiments were conducted in 384-well plates. Immediately after the addition of Aβ (final concentration of 20 μM), time traces were recorded using a Varioskan plate reader (ThermoFisher, Waltham, MA) with excitation at 440 nm (λ_ex_) and emission at 480 nm (λ_em_). The readings were taken at 37 °C with the plate being shaken at 600 rpm and a 1 mm diameter of orbital movement for 10 s before each reading.

### Proteasome assay

The chymotryptic like (ChT-L) activity of the 20S proteasome in the presence of AloA and AloB and their equimolar mixtures AloAB, was tested by incubating the compounds (1–750 μM) with the 20S core particle at a final concentration of 1.3 nM, in 50 mM Tris–HCl (pH 7.4) at 37 °C for max 45 min. Then the fluorogenic peptide Suc-LLVY-AMC was added at 100 μM and the fluorescence intensity at 440 (excitation at 360 nm) was monitored to follow the release of aminomethylcoumarin (AMC). The reaction volume of every reaction was 50 μL and the well fluorescence were analyzed by Victor (Perkin Elmer) plate reader, in a 384-well plate. A minimum of three replicates were performed for each data point. The slope of the emission of AMC at 440 nm can be calculated and it is correlated to proteasome activity. The obtained data were normalized with respect to the control experiment in the absence of a tested compound that was regarded as 100%. The potency (IC_50_) was calculated from the value of the dose–response plot corresponding to a fractional activity (%v) of 50%. The IC_50_ values were determined using a non-linear fit of experimental data according to the following equation where X is the concentration:

$$\%v=100/\left(1+{\left(\frac{IC50}{X}\right)}^{HillSlope}\right).$$

### Circular dichroism

Circular dichroism (CD) measurements were recorded at a temperature of 4 °C using a Jasco J-810 spectropolarimeter. Quartz cuvettes with a 1 cm optical path were employed, and the measurements were carried out in the 200–450 nm wavelength range. The reported spectra were derived by routinely subtracting the ligand spectrum, which had been recorded under the same experimental conditions.

### Cell cultures

The human neuroblastoma cell lines, SH-SY5Y, were maintained in DMEM-F12 (Gibco, Thermofisher) supplemented with 10% heat-inactivated (HI) fetal bovine serum (Gibco, Thermofisher), 100 mg/mL penicillin and streptomycin (Gibco, Thermofisher), and 2 mM l-glutamine at 37 °C, 5% CO_2_.

HeLa Cells were maintained in DMEM (Gibco, Thermofisher) supplemented with 10% heat-inactivated (HI) fetal bovine serum (Gibco, Thermofisher), 100 mg/mL penicillin and streptomycin (Gibco, Thermofisher), and 2 mM l-glutamine at 37 °C, 5% CO_2_.

### Aloin anti-proliferative assay

One day before experiments, 2.5 × 10^4^ cells/well were seeded on a 96-multiwell plate in freshly prepared DMEM-F12 with 5% heat-inactivated (HI) fetal calf serum. Real-time cell proliferation data were collected using the Sartorius IncuCyte® SX1 system that was placed inside a cell culture incubator. Before treatments, cells were washed twice with phosphate buffer saline (PBS) and the medium was replaced with freshly prepared DMEM-F12 supplemented with 3% heat-inactivated (HI) fetal calf serum. Cells were exposed to increasing concentrations of AloA and AloB (0-400 µM) and plates were incubated for 48 h at 37 °C and 5% CO_2_. Untreated cells were used as control. Images were collected every 6 h for 48 h, using a 10× objective. A readout of cytotoxicity over time was obtained by IncuCyte® SX1 Live-Cell Analysis Software.

### MTT assay

To test the cytotoxicity of Aloin, SH-SY5Y, and HeLa cells were seeded at 2.5 × 10^4^ and 1 × 10^4^ cells/well respectively in a 96 multi-well plate. Before the experiment, cells were washed twice with PBS and the medium was replaced with freshly prepared DMEM-F12 or DMEM supplemented with 3% heat-inactivated (HI) fetal calf serum. Cells were treated with increasing concentrations of AloA, AloB, AloAB (0–1 mM), and bortezomib (0–100 nM) used as positive control.

To test the activity of loaded AloAB onto CPDs-PNM, cells were exposed to 0–100 μM of free and bounded AloAB. In both experiments, we used untreated cells as a control. After 48 h treatment, cell cultures were incubated with MTT (0.5 mg/mL) for 2 h at 37 °C and then lysed with DMSO; the formazan production was evaluated in a plate reader through the absorbance at 570 nm, using the Victor Nivo, PerkinElmer® multiwell plate reader.

### AloAB loading

AloAB (1.2 mg) was added to a solution of CDs-PNM (1.1 mg/mL) in water, followed by stirring at room temperature for 72 h in the absence of light. The yellowish dispersion was purified by dialysis using Spectra/Por membrane 3–5 kDa cut off MWCO upon to absence of drug in the dialysis medium. The amount of AloAB loaded in the CDs-PNM nanostructures was estimated by optical absorption measurements. The drug loading capacity (LC, %) was calculated by the following equation:

$$LC\left(\%\right)=\frac{mg\, drug \,in}{mg\, Cdots +mg \,drug\, in }.$$

### Compliance statement regarding research on plants

The extraction of the two metabolites was carried out from *Aloe arborescens* leaves, a species not at risk of extinction. Therefore, the supply, extraction and recovery methods are not subject to any legislative/institutional authorization or guideline.

### Supplementary Information

Supplementary Information.

## Data Availability

The datasets used and/or analysed during the current study are available from the corresponding author on reasonable request.

## References

[1] Bagewadi, H. G., Patil, B. V. & Zahid, S. H. Rotarod test and Catalepsy bar test: Behavioral testing and neuromodulation of Aloe vera in MPTP induced Parkinson’s disease animal model. *Int. J. Basic Clin. Pharmacol.* **7**, 494. 10.18203/2319-2003.ijbcp20180663 (2018). 10.18203/2319-2003.ijbcp20180663

[2] Luo, X. *et al.* Aloin suppresses lipopolysaccharide-induced inflammatory response and apoptosis by inhibiting the activation of NF-κB. *Molecules* **23**, 517 (2018). 29495390 10.3390/molecules23030517 PMC6017010

[3] Radha, M. H. & Laxmipriya, N. P. Evaluation of biological properties and clinical effectiveness of Aloe vera: A systematic review. *J. Tradit. Complement. Med.* **5**, 21–26 (2015). 26151005 10.1016/j.jtcme.2014.10.006 PMC4488101

[4] Rana, M. N. *et al.**Thunbergia laurifolia* leaf extract partially recovers lead-induced renotoxicity through modulating the cell signaling pathways. *Saudi J. Biol. Sci.* **27**, 3700–3710 (2020). 34466056 10.1016/j.sjbs.2020.08.016 PMC8381871

[5] Salehi, B. *et al.* Aloe genus plants: From farm to food applications and phytopharmacotherapy. *Int. J. Mol. Sci.* **19**, 2843 (2018). 30235891 10.3390/ijms19092843 PMC6163315

[6] Sharma, A. K., Khanna, D. & Balakumar, P. Low-dose dipyridamole treatment partially prevents diabetes mellitus-induced vascular endothelial and renal abnormalities in rats. *Int. J. Cardiol.* **172**, 530–532. 10.1016/j.ijcard.2014.01.053 (2014). 24495652 10.1016/j.ijcard.2014.01.053

[7] Svitina, H., Hamman, J. & Gouws, C. Molecular mechanisms and associated cell signalling pathways underlying the anticancer properties of phytochemical compounds from *Aloe* species (Review). *Exp. Ther. Med.* **22**, 852. 10.3892/etm.2021.10284 (2021). 34178125 10.3892/etm.2021.10284 PMC8220653

[8] Maiti, P. *et al.* Tetrahydrocurcumin has similar anti-amyloid properties as curcumin: In vitro comparative structure-activity studies. *Antioxidants* **10**, 1592 (2021). 34679727 10.3390/antiox10101592 PMC8533373

[9] Sharma, D. *et al.* A review on pharmacological and therapeutic potential of *Aloe barbadensis* Miller. *Eur. J. Med. Plants* **33**, 23–43. 10.9734/EJMP/2022/v33i630471 (2022). 10.9734/EJMP/2022/v33i630471

[10] Duan, W. *et al.* New role of JAK2/STAT3 signaling in endothelial cell oxidative stress injury and protective effect of melatonin. *PLoS ONE* **8**, e57941 (2013). 23483946 10.1371/journal.pone.0057941 PMC3590213

[11] Gouveia, G. R. *et al.* Overexpression of OCT-1 gene is a biomarker of adverse prognosis for diffuse large B-cell lymphoma (DLBCL): Data from a retrospective cohort of 77 Brazilian patients. *BMC Cancer* **20**, 1041. 10.1186/s12885-020-07553-2 (2020). 33121489 10.1186/s12885-020-07553-2 PMC7596969

[12] Mohamed Naveed, J. F. *et al.* Phytochemical study in ethanolic leaves extract of Aloe vera using gas chromatography. *Int. J. Res. Pharm. Sci.* **10**, 1470–1473. 10.26452/ijrps.v10i2.720 (2019). 10.26452/ijrps.v10i2.720

[13] Manitto, P., Monti, D. & Speranza, G. Studies on aloe. Part 6. Conformation and absolute configuration of Aloins A and B and related 10-C-glucosyl-9-anthrones. *J. Chem. Soc. Perkin Trans.* **1**, 1297–1300. 10.1039/P19900001297 (1990). 10.1039/P19900001297

[14] Lanouar, F., Bougattass, I., Bousserhine, N. & Banni, M. Biochemical and transcriptomic evaluation of the toxic effects of Aloin contaminated agricultural soils on the earth worm *Eisenia andrei*. *Int. J. Environ. Agric. Biotechnol.* **3**, 239119 (2018).

[15] Solaberrieta, I., Jiménez, A. & Garrigós, M. C. Valorization of aloe vera skin by-products to obtain bioactive compounds by microwave-assisted extraction: Antioxidant activity and chemical composition. *Antioxidants* **11**, 1058 (2022). 35739955 10.3390/antiox11061058 PMC9220353

[16] Peña, J. S. & Vazquez, M. Harnessing the neuroprotective behaviors of Müller glia for retinal repair. *Front. Biosci. (Landmark Ed.)* **27**, 169 (2022). 35748245 10.31083/j.fbl2706169 PMC9639582

[17] Li, X. *et al.* Using N-doped carbon dots prepared rapidly by microwave digestion as nanoprobes and nanocatalysts for fluorescence determination of ultratrace isocarbophos with label-free aptamers. *Nanomaterials* **9**, 223 (2019). 30736465 10.3390/nano9020223 PMC6409902

[18] Sun, Y.-P. *et al.* Host-guest carbon dots for enhanced optical properties and beyond. *Sci. Rep.* **5**, 12354 (2015). 26196598 10.1038/srep12354 PMC4508828

[19] Sadiq, U., Gill, H. & Chandrapala, J. Temperature and pH stability of Anthraquinones from native aloe vera gel, spray-dried and freeze-dried aloe vera powders during storage. *Foods* **11**, 1613. 10.3390/foods11111613 (2022). 35681363 10.3390/foods11111613 PMC9180388

[20] Ding, W.-J., Wu, X.-F., Zhong, J.-S. & Wan, J.-Z. Effects of temperature, pH and light on the stability of Aloin A and characterisation of its major degradation products. *Int. J. Food Sci. Technol.* **49**, 1773–1779. 10.1111/ijfs.12500 (2014). 10.1111/ijfs.12500

[21] Selkoe, D. J. The molecular pathology of Alzheimer’s disease. *Neuron* **6**, 487–498 (1991). 1673054 10.1016/0896-6273(91)90052-2

[22] Coria, F., Rubio, I. & Bayon, C. Alzheimer’s disease, ß-amyloidosis, and aging. *Rev. Neurosci.* **5**, 275–292. 10.1515/REVNEURO.1994.5.4.275 (1994). 7697197 10.1515/REVNEURO.1994.5.4.275

[23] Hamley, I. W. The amyloid beta peptide: A chemist’s perspective. Role in Alzheimer’s and fibrillization. *Chem. Rev.* **112**, 5147–5192. 10.1021/cr3000994 (2012). 22813427 10.1021/cr3000994

[24] Hyung, S.-J. *et al.* Insights into antiamyloidogenic properties of the green tea extract (−)-epigallocatechin-3-gallate toward metal-associated amyloid-β species. *Proc. Natl. Acad. Sci.* **110**, 3743–3748. 10.1073/pnas.1220326110 (2013). 23426629 10.1073/pnas.1220326110 PMC3593904

[25] Ahmed, R. *et al.* Atomic resolution map of the soluble amyloid beta assembly toxic surfaces. *Chem. Sci.* **10**, 6072–6082. 10.1039/C9SC01331H (2019). 31360412 10.1039/C9SC01331H PMC6585597

[26] Lolicato, F., Raudino, A., Milardi, D. & La Rosa, C. Resveratrol interferes with the aggregation of membrane-bound human-IAPP: A molecular dynamics study. *Eur. J. Med. Chem.* **92**, 876–881 (2015). 25638571 10.1016/j.ejmech.2015.01.047

[27] Romanucci, V. *et al.* Modulating Aβ aggregation by tyrosol-based ligands: The crucial role of the catechol moiety. *Biophys. Chem.* **265**, 106434. 10.1016/j.bpc.2020.106434 (2020). 32707474 10.1016/j.bpc.2020.106434

[28] Sciacca, M. F. *et al.* Inhibition of Aβ amyloid growth and toxicity by silybins: The crucial role of stereochemistry. *ACS Chem. Neurosci.* **8**, 1767–1778 (2017). 28562008 10.1021/acschemneuro.7b00110

[29] Hough, R., Pratt, G. & Rechsteiner, M. Ubiquitin-lysozyme conjugates. Identification and characterization of an ATP-dependent protease from rabbit reticulocyte lysates. *J. Biol. Chem.* **261**, 2400–2408 (1986). 3003114 10.1016/S0021-9258(17)35950-1

[30] Voges, D., Zwickl, P. & Baumeister, W. The 26S proteasome: A molecular machine designed for controlled proteolysis. *Annu. Rev. Biochem.* **68**, 1015–1068. 10.1146/annurev.biochem.68.1.1015 (1999). 10872471 10.1146/annurev.biochem.68.1.1015

[31] Harris, J. R. Release of a macromolecular protein component from human erythrocyte ghosts. *Biochim. Biophys. Acta* **150**, 534–537 (1968). 5650402 10.1016/0005-2736(68)90157-0

[32] Hegerl, R. *et al.* The three-dimensional structure of proteasomes from *Thermoplasma acidophilum* as determined by electron microscopy using random conical tilting. *FEBS Lett.* **283**, 117–121 (1991). 2037064 10.1016/0014-5793(91)80567-M

[33] Orlowski, M. & Wilk, S. Catalytic activities of the 20 S proteasome, a multicatalytic proteinase complex. *Arch. Biochem. Biophys.* **383**, 1–16. 10.1006/abbi.2000.2036 (2000). 11097171 10.1006/abbi.2000.2036

[34] Adams, J. The proteasome: Structure, function, and role in the cell. *Cancer Treat. Rev.* **29**, 3–9. 10.1016/S0305-7372(03)00081-1 (2003). 12738238 10.1016/S0305-7372(03)00081-1

[35] Arlt, A. *et al.* Increased proteasome subunit protein expression and proteasome activity in colon cancer relate to an enhanced activation of nuclear factor E2-related factor 2 (Nrf2). *Oncogene* **28**, 3983–3996. 10.1038/onc.2009.264 (2009). 19734940 10.1038/onc.2009.264

[36] Chen, D. *et al.* Dietary flavonoids as proteasome inhibitors and apoptosis inducers in human leukemia cells. *Biochem. Pharmacol.* **69**, 1421–1432. 10.1016/j.bcp.2005.02.022 (2005). 15857606 10.1016/j.bcp.2005.02.022

[37] Kumatori, A. *et al.* Abnormally high expression of proteasomes in human leukemic cells. *Proc. Natl. Acad. Sci.* **87**, 7071–7075. 10.1073/pnas.87.18.7071 (1990). 2205851 10.1073/pnas.87.18.7071 PMC54685

[38] Roeten, M. S. F., Cloos, J. & Jansen, G. Positioning of proteasome inhibitors in therapy of solid malignancies. *Cancer Chemother. Pharmacol.* **81**, 227–243. 10.1007/s00280-017-3489-0 (2018). 29184971 10.1007/s00280-017-3489-0 PMC5778165

[39] Chang, S. C. & Ding, J. L. Ubiquitination and SUMOylation in the chronic inflammatory tumor microenvironment. *Biochim. Biophys. Acta Rev. Cancer* **1870**, 165–175. 10.1016/j.bbcan.2018.08.002 (2018). 30318471 10.1016/j.bbcan.2018.08.002

[40] Kaplan, G. S. *et al.* Proteasome inhibitors in cancer therapy: Treatment regimen and peripheral neuropathy as a side effect. *Free Radic. Biol. Med.* **103**, 1–13. 10.1016/j.freeradbiomed.2016.12.007 (2017). 27940347 10.1016/j.freeradbiomed.2016.12.007

[41] Ogiso, Y., Tomida, A., Kim, H.-D. & Tsuruo, T. Glucose starvation and hypoxia induce nuclear accumulation of proteasome in cancer cells. *Biochem. Biophys. Res. Commun.* **258**, 448–452. 10.1006/bbrc.1999.0635 (1999). 10329407 10.1006/bbrc.1999.0635

[42] Santoro, A. M. *et al.* Cationic porphyrins are tunable gatekeepers of the 20S proteasome. *Chem. Sci.* **7**, 1286–1297. 10.1039/C5SC03312H (2016). 10.1039/c5sc03312h PMC5975898 29910886

[43] Consoli, G. M. L. *et al.* A novel facile one-pot synthesis of photothermally responsive carbon polymer dots as promising drug nanocarriers. *Chem. Commun.* **58**, 3126–3129 (2022). 10.1039/d1cc06530k 35018398

[44] Consoli, G. M. L. *et al.* Green Light-Triggerable Chemo-Photothermal Activity of Cytarabine-Loaded Polymer Carbon Dots: Mechanism and Preliminary In Vitro Evaluation. *ACS Appl. Mater. Interfaces* **15**, 5732–5743. 10.1021/acsami.2c22500 (2023) 10.1021/acsami.2c22500 PMC9906628 36688816

[45] Consoli, G. M. *et al.* Red light-triggerable nanohybrids of graphene oxide, gold nanoparticles and thermo-responsive polymers for combined photothermia and drug release effects. *J. Mater. Chem. B* **12**, 952–961 (2024) 10.1039/d3tb01863f 37975827

[46] Consoli, G. M. L. Forte, G. Maugeri, L. & Petralia, S. Photo‐Responsive TiO 2 ‐Gold Nanoparticle‐Polymer Nanohybrid Exhibits Photothermal, Thermo‐Release, and Photocatalytic Effects. *Chem. Photo. Chem.* e202400088. 10.1002/cptc.202400088 (2024).

[47] Forte, G. *et al.* A nanosized photothermal responsive core-shell carbonized polymer dots based on poly (N-isopropylacrylamide) for light-triggered drug release. *Colloid. Surf. B: Biointer.* **217**, 112628 (2022). 10.1016/j.colsurfb.2022.112628 35716451

[48] Tabolacci, C. *et al.* Aloin enhances cisplatin antineoplastic activity in B16-F10 melanoma cells by transglutaminase-induced differentiation. *Amino Acids* 44, 293–300. 10.1007/s00726-011-1166-x (2013). 10.1007/s00726-011-1166-x 22139409

[49] Esmat, A. Y., Tomasetto, C. & Rio, M.-C. Cytotoxicity of a natural anthraquinone (Aloin) against human breast cancer cell lines with and without ErbB-2: Topoisomerase II-alpha coamplification. *Cancer Biol. Therapy* **5**, 97–103. 10.4161/cbt.5.1.2347 (2006). 10.4161/cbt.5.1.2347 16357514

[50] Hayes, A. W., & Clemens, R. A. & Pressman, P. The absence of genotoxicity of a mixture of aloin A and B and a commercial aloe gel beverage. *Toxicol. Mech. Methods* **32**, 385–394. 10.1080/15376516.2021.2023828 (2022). 10.1080/15376516.2021.2023828 34979868

