# Supplementary material for: Exploring the therapeutic potential of Aloin: unraveling neuroprotective and anticancer mechanisms, and strategies for enhanced stability and delivery
Source: Sci Rep. 2024 Jul 20;14:16731. doi: 10.1038/s41598-024-67397-9 (PMC11271566; doi:10.1038/s41598-024-67397-9)
Supplement: Supplementary file 1 — Supplementary Information. [file 41598_2024_67397_MOESM1_ESM.docx]

**Exploring the Therapeutic Potential of Aloin: Unraveling Neuroprotective and Anticancer Mechanisms, and Strategies for Enhanced Stability and Delivery.**

**Supporting Information**

Supporting Information

**Table of contents** *pag.*

1. ^1^ H, ^13^C and 2D NMR data of Aloin A and Aloin B (Table **1S** and **2S**)
2. HPLC profiles of Aloin A and Aloin B (Figure **1S** and **2S**)
3. LC-MS analysis of Aloin A and Aloin B at t = 0 and t = 24 h. (Figure **3S** and **4S**)
4. Circular Dichroism spectra of aloin-loaded Carbon dots (Figure **5S**)
5. Antiproliferative assays on HeLa cells (Figures **6S**-**8S**)

**Table S1**. ^1^ H, ^13^C and 2D NMR data of **Aloin A** in CD_3_OH.

| Position | Residue | ^13^C^a^ | ^1^H^a^ , multiplicity  (J in Hz) | ^1^H-^1^H COSY | ^1^H-^13^C HMBC |
| --- | --- | --- | --- | --- | --- |
| 1 | C | 162.02 | - |  |  |
| 2 | CH | 117.69 | 7.07, s |  | 162.02, 116.33, 113.00, 63.11 |
| 3 | C | 150.15 | - |  |  |
| 4 | CH | 113.00 | 6.90, s |  | 117.69, 116.33, 63.11, 44.54 |
| 5 | CH | 118.54 | 7.08, d  (8.2) | 7.52 | 117.27, 115.42, 44.54 |
| 6 | CH | 135.62 | 7.52, t  (8.2) | 7.08, 6.88 | 161.57, 145.18 |
| 7 | CH | 115.42 | 6.88, d  (8.4) | 7.52 | 118.54, 117.27 |
| 8 | C | 161.57 | - |  |  |
| 9 | C | 194.15 | - |  |  |
| 10 | CH | 44.54 | 4.63, s | 3.43 | 118.54, 117.27, 116.33, 113.00, 85.24 |
| 11 | CH_2_ | 63.11 | 4.70, d  (14.6)  4.66, d  (14.6) | 4.66  4.70 | 150.15, 117.69, 113.00 |
| 1a | C | 116.33 | - |  |  |
| 4a | C | 141.90 | - |  |  |
| 5a | C | 145.18 | - |  |  |
| 8a | C | 117.27 | - |  |  |
| 1’ | CH | 85.24 | 3.43, dd  (11.4, 1.8) | 4.63, 3.03 | 145.18, 141.90, 80.31, 78.58, 44.54 |
| 2’ | CH | 70.46 | 3.03, t  (9.4) | 3.43, 3.27 | 70.60, 44.54 |
| 3’ | CH | 78.58 | 3.27, t  (8.7) | 3.03, 2.92 | 85.24, 80.31 |
| 4’ | CH | 70.60 | 2.92, t  (9.5) | 3.27, 2.94 | 70.46, 61.84 |
| 5’ | CH | 80.31 | 2.94, m | 3.58, 3.40, 2.92 | 85.24, 78.58, 61.84 |
| 6’ | CH_2_ | 61.84 | 3.58, dd  (11.2, 1.9)  3.40, dd  (11.2, 5.2) | 3.40, 2.94  3.58, 2.94 | 80.31, 70.60  80.31, 70.60 |

^a^Chemical shifts in ppm.

**Table S2**. ^1^ H, ^13^C and 2D NMR data of **Aloin B** in CD_3_OH.

| Position | Residue | ^13^C^a^ | ^1^H^a^ , multiplicity  (J in Hz) | ^1^H-^1^H COSY | ^1^H-^13^C HMBC |
| --- | --- | --- | --- | --- | --- |
| 1 | C | 161.80 | - |  |  |
| 2 | CH | 116.31 | 7.09, s |  | 161.80, 116.17, 112.74, 63.09 |
| 3 | C | 150.97 | - |  |  |
| 4 | CH | 112.74 | 6.90, s |  | 116.17, 116.31, 63.09, 44.47 |
| 5 | CH | 119.86 | 7.11, d  (8.4) | 7.51 | 117.54, 115.71, 44.47 |
| 6 | CH | 134.86 | 7.51, dd  (8.8, 7.6) | 7.11, 6.89 | 161.77, 145.44 |
| 7 | CH | 115.71 | 6.89, dd  (6.8, 1.1) | 7.51 | 119.86, 117.54 |
| 8 | C | 161.77 | - |  |  |
| 9 | C | 194.17 | - |  |  |
| 10 | CH | 44.47 | 4.65, d  (1.9) | 3.44 | 119.86, 117.54, 116.17, 112.74, 85.20 |
| 11 | CH_2_ | 63.09 | 4.68, d  (15.5)  4.64, d  (15.5) |  | 150.97, 116.31, 112.74 |
| 1a | C | 116.17 | - |  |  |
| 4a | C | 141.68 | - |  |  |
| 5a | C | 145.44 | - |  |  |
| 8a | C | 117.54 | - |  |  |
| 1’ | CH | 85.20 | 3.44, dd  (9.8, 2.2) | 4.65, 3.06 | 145.44, 141.68, 80.23, 78.56, 44.47 |
| 2’ | CH | 70.46 | 3.06, t  (9.8) | 3.44, 3.28 | 70.59, 44.47 |
| 3’ | CH | 78.56 | 3.28, t  (9.0) | 3.06, 2.93 | 85.20, 80.23 |
| 4’ | CH | 70.59 | 2.93, m | 3.28, 2.94 | 70.46, 61.84 |
| 5’ | CH | 80.23 | 2.94, m | 3.58, 3.41, 2.93 | 85.20, 78.56, 61.84 |
| 6’ | CH_2_ | 61.84 | 3.58, dd  (11.2, 1.7)  3.41, dd  (11.2, 4.6) | 3.41, 2.94  3.58, 2.94 | 80.23, 70.59  80.23, 70.59 |

^a^Chemical shifts in ppm.


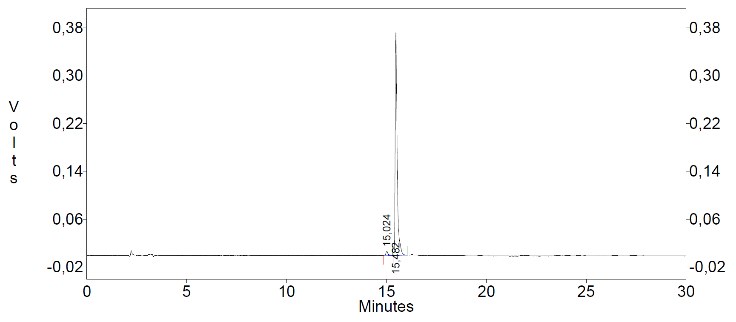


**A)**

**B)**


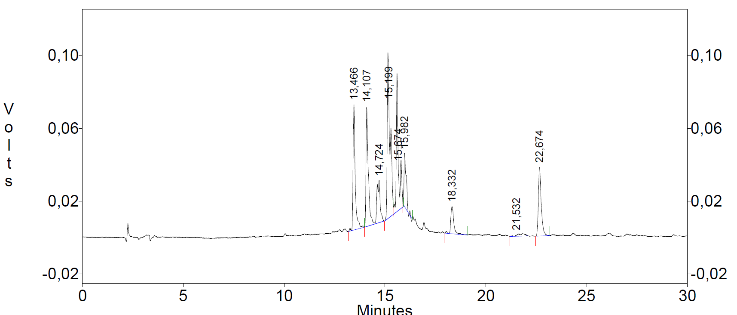


**Figure S1** HPLC profiles of Aloin A in PBS at pH = 7.4, time 0 (**A**) and after 24h at 37°C (**B**).


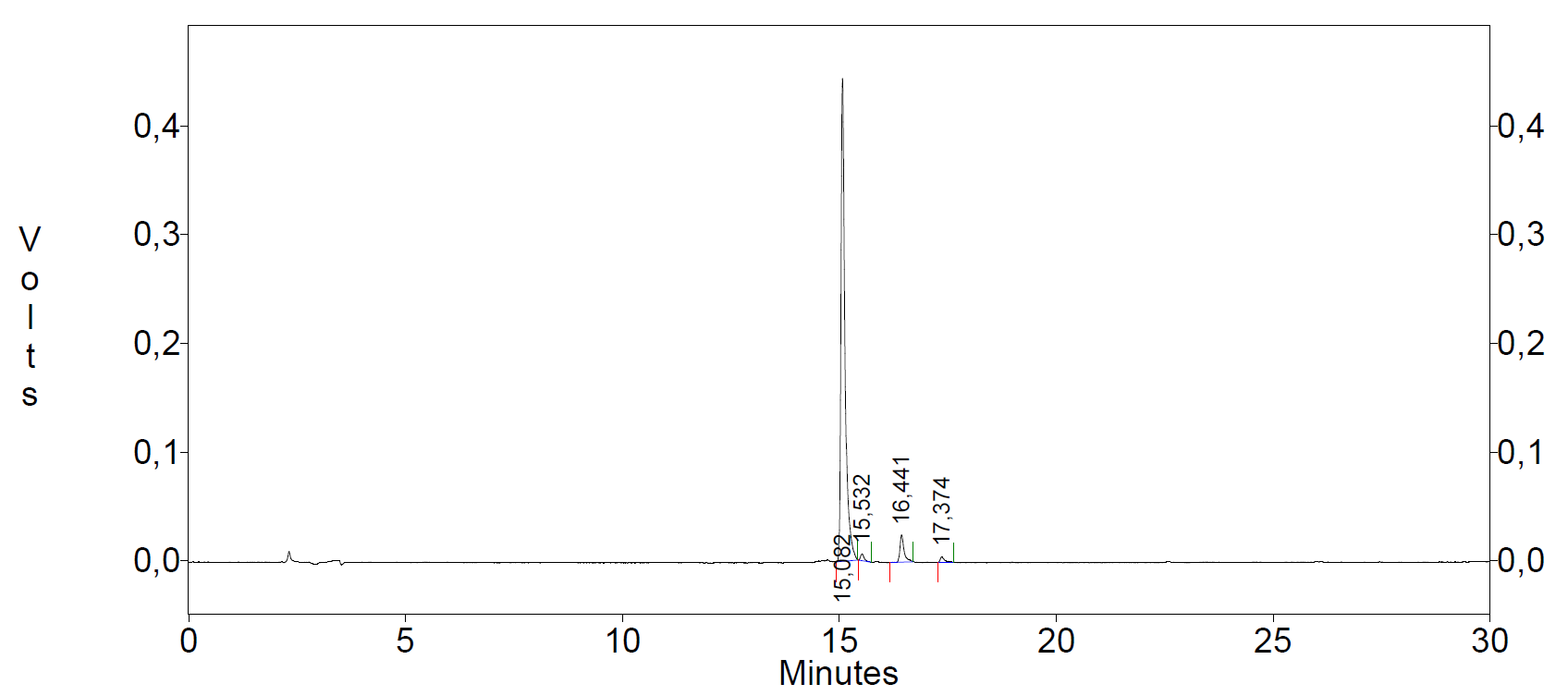


**A)**

**B)**


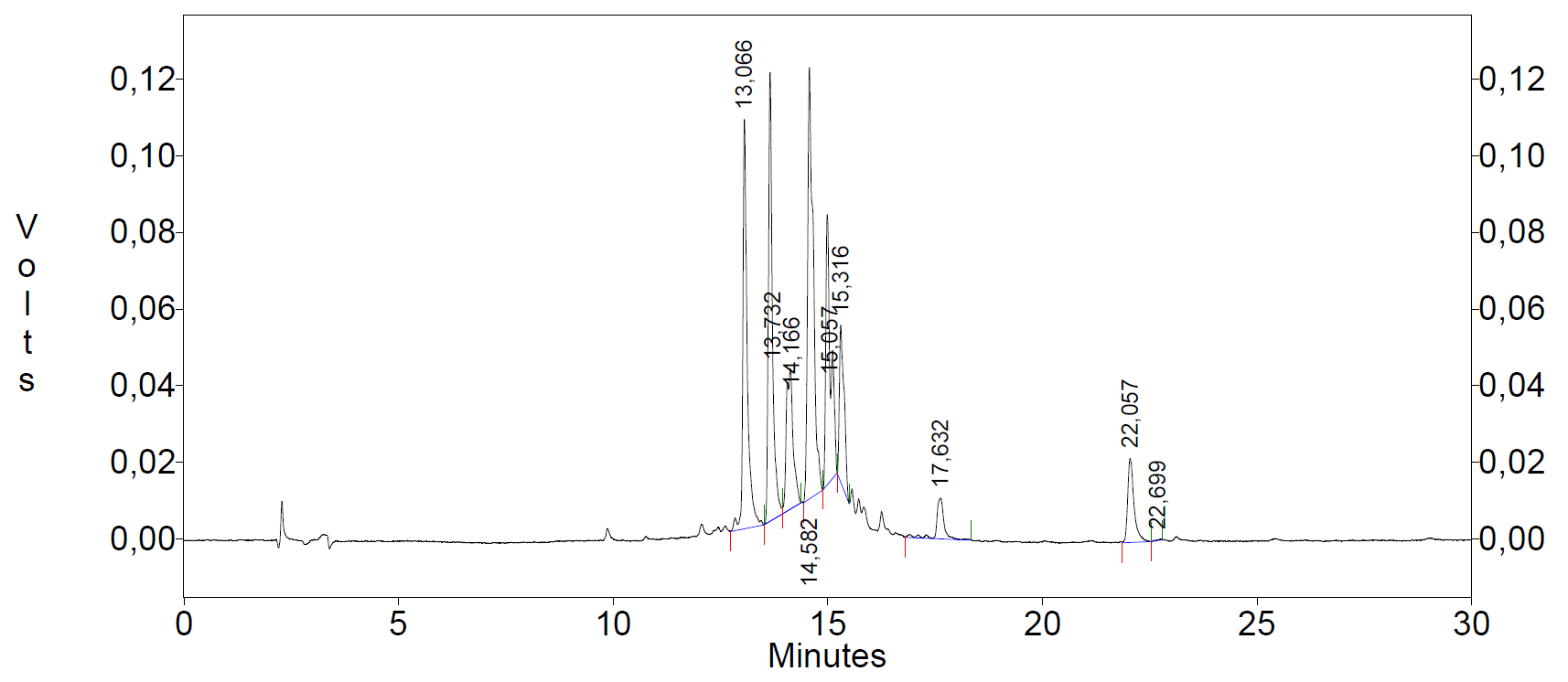


**Figure S2** HPLC profiles of Aloin B at rt, in PBS at pH = 7.4, time 0 (**A**) and after 24h at 37°C (**B**).

#


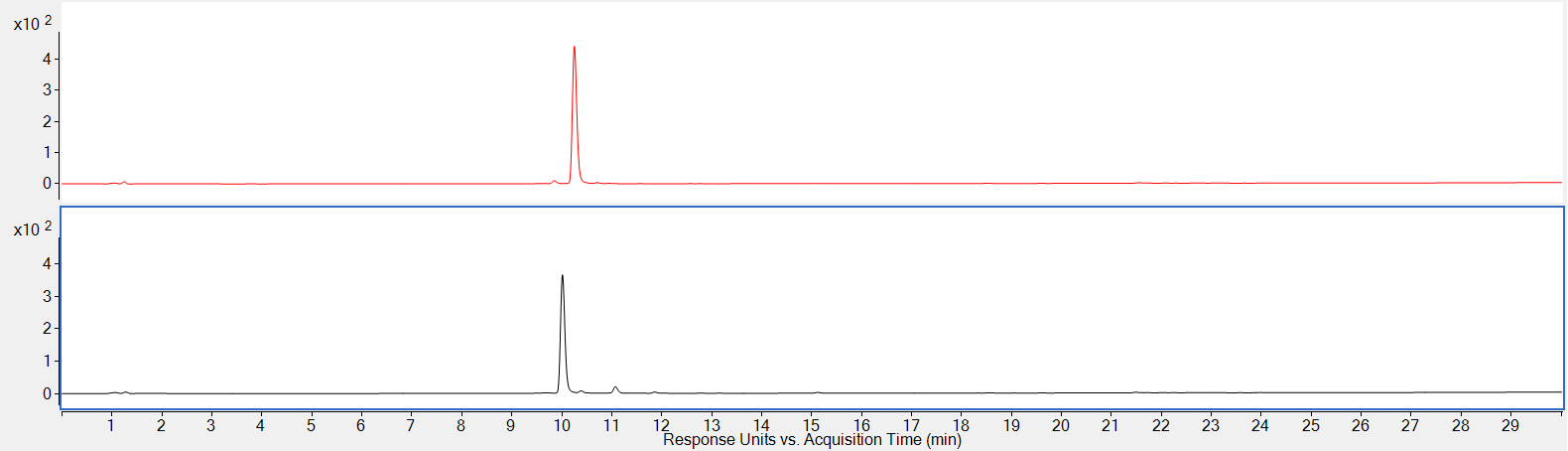


**A)**

**B)**

*


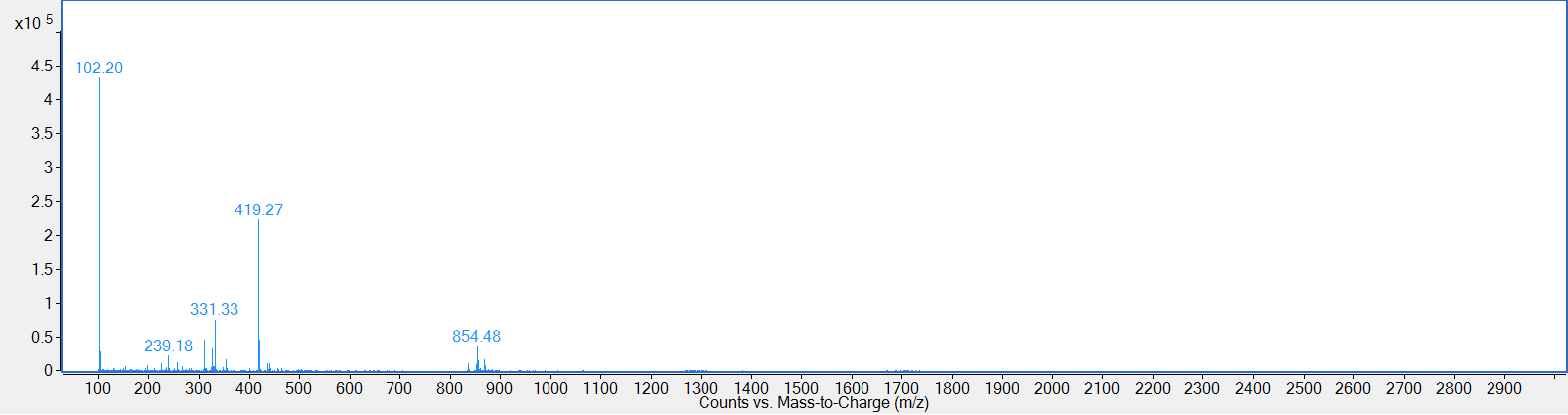


Aloin

[M] = 418.39

[MH]^+^

**Figure S3.** LC-MS analysis: (**A**) Chromatograms composed of total ion current chromatogram (TIC) of Aloin A (red line) and Aloin B (black line) at rt, in PBS at pH = 7.4, at time 0; (**B**) MS spectrum (positive mode) of peaks at t*_R_* = 10.1 min. (*) and t*_R_* = 10.2 min. (#)


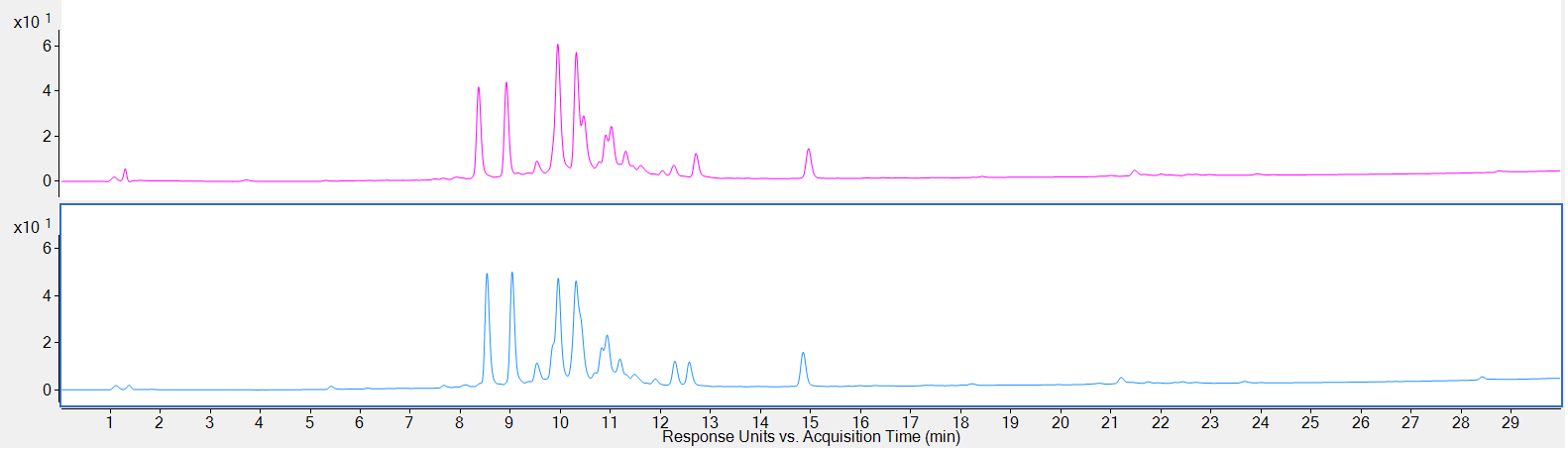


**A)**

**B)**

#

*

#

*


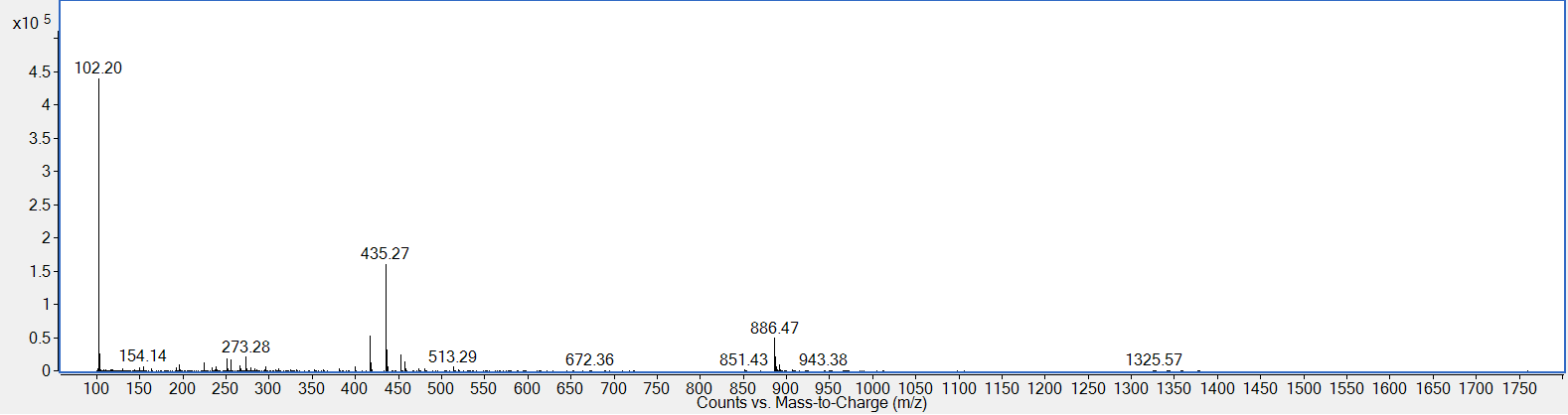


Aloin-10-OH

[M] = 434.39

[MH]^+^

**Figure S4.** LC-MS analysis: (**A**) Chromatograms composed of total ion current chromatogram (TIC) of Aloin A (magenta line) and Aloin B (blue line) at 37°C, in PBS at pH = 7.4, at 24h; (**B**) MS spectrum (positive mode) of peak at t*_R_* = 8.6 min. (*) and t*_R_* = 9.1 min (#).


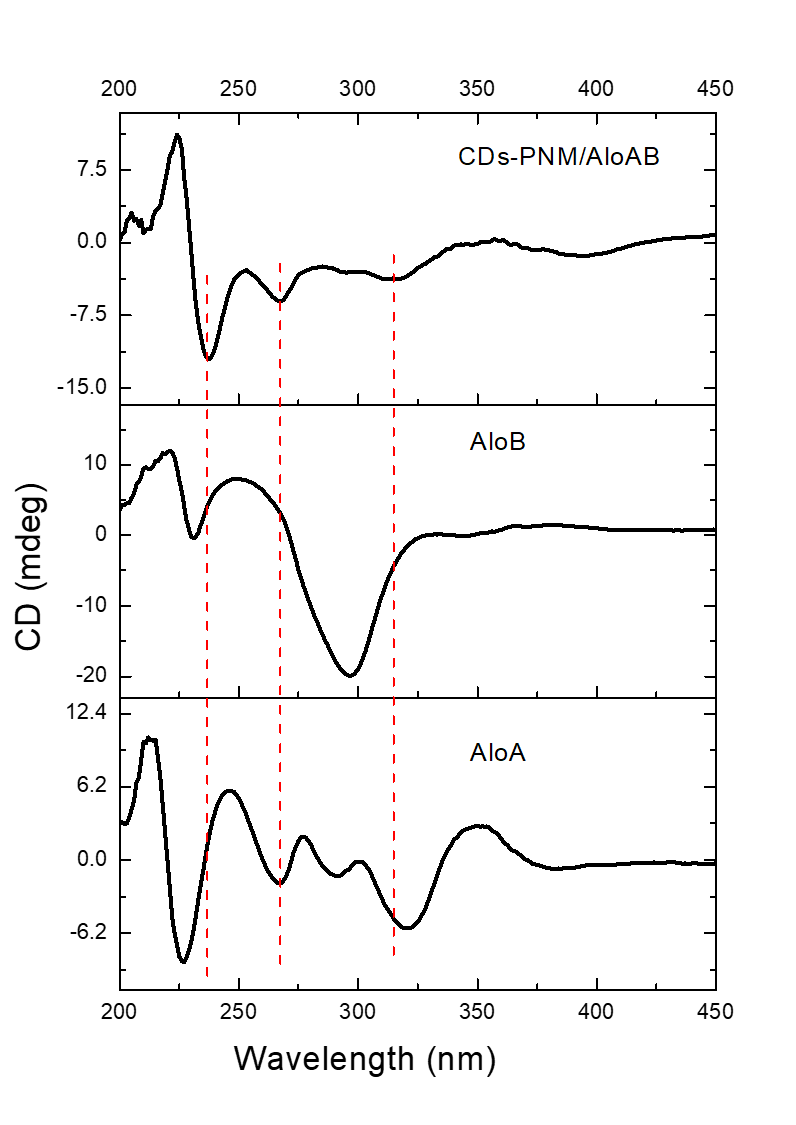


**Figure S5.** CDs spectra of CDs-PNM/AloAB, AloA and AloB.

**A) B)**


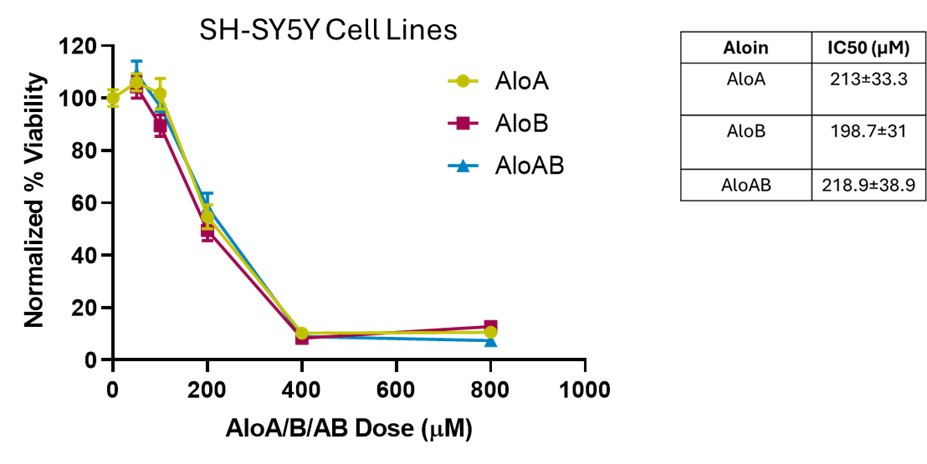


**Figure S6.** Effect of 48 hours exposure of neuroblastoma cell line to Aloin. A) Dose-Response curves of AloA AloB, AloAB (50, 100, 200, 400, 800 µM) measured by MTT Assay. B) IC50 values of AloA, AloB, AloAB. Values represent mean ±SEM of three independent experiments with n=3 each.


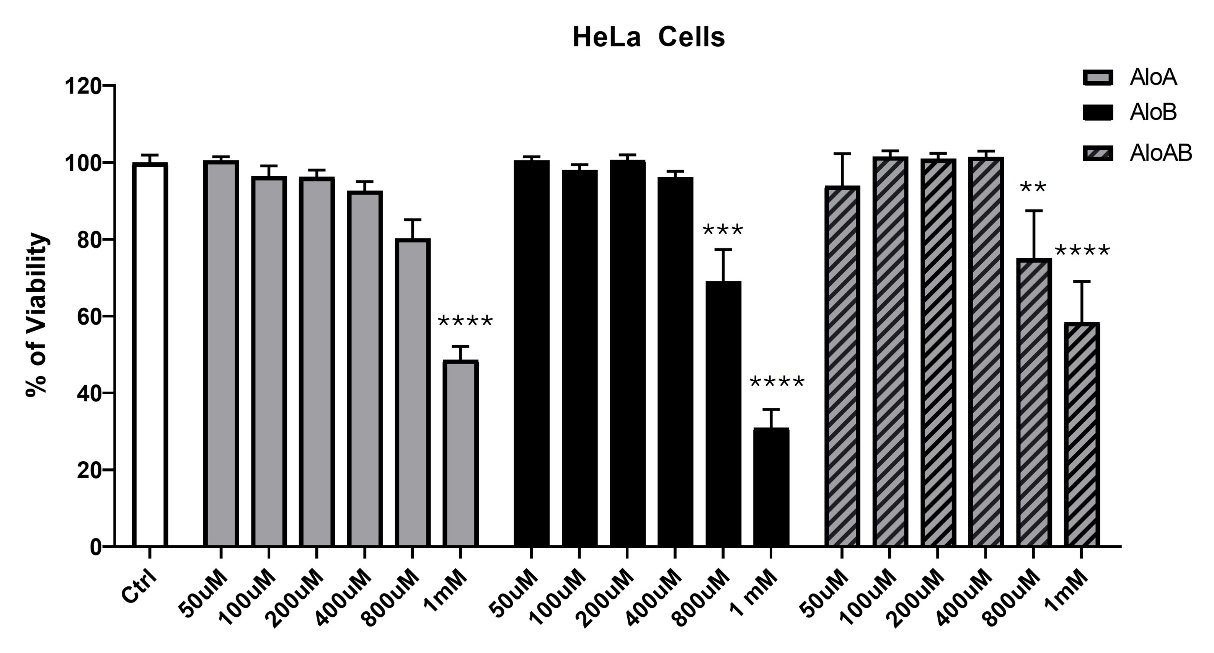


**Figure S7.** MTT Assay of HeLa cells treated for 48 hours with increasing concentrations of AloA AloB, AloAB (50µM-1mM). Bars represent mean ±SEM of three independent experiments with n=3 each. ****P<0.0001 vs Ctrl by One-Way ANOVA + Dunnett’s Test.

**A) B)**


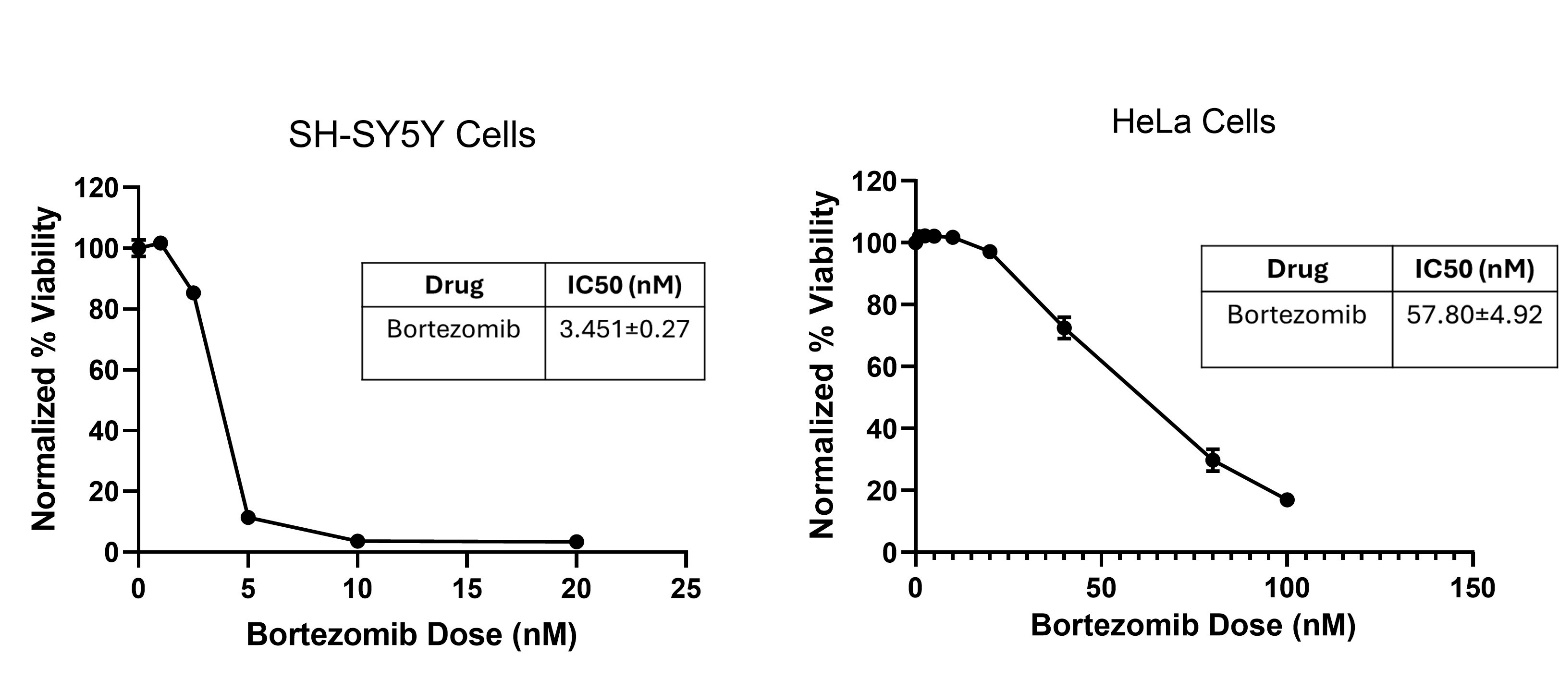


**Figure S8.** Effect of 48 hours exposure of Neuroblastoma and HeLa cell line to Bortezomib. A) Dose-response curve of SH-SY5Y (1, 2.5, 5, 10, and 20 nM) and B) dose-response curve of HeLa cell line (1, 2.5, 5, 10, 20, 40, 80, 100 nM) measured by MTT Assay. Values represent mean ±SEM of three independent experiments with n=3 each.
